# Body fatness, diabetes, physical activity and risk of kidney stones: a systematic review and meta-analysis of cohort studies

**DOI:** 10.1007/s10654-018-0426-4

**Published:** 2018-07-31

**Authors:** Dagfinn Aune, Yahya Mahamat-Saleh, Teresa Norat, Elio Riboli

**Affiliations:** 1 Department of Epidemiology and Biostatistics, School of Public Health, Imperial College London, St. Mary’s Campus, Norfolk Place, Paddington, London, W2 1PG UK; 2 Department of Nutrition, Bjørknes University College, Oslo, Norway; 3 Department of Endocrinology, Morbid Obesity and Preventive Medicine, Oslo University Hospital, Oslo, Norway; 4 INSERM (French National Institute for Health and Medical Research), CESP, Gustave Roussy, Health Across Generations Team, Villejuif, France

**Keywords:** Body mass index, Waist circumference, Waist-to-hip ratio, Diabetes, Physical activity, Kidney stones, Systematic review, Meta-analysis, Cohort studies

## Abstract

We conducted a systematic review and meta-analysis to clarify the association between adiposity, diabetes, and physical activity and the risk of kidney stones. PubMed and Embase were searched up to April 22nd 2018 for relevant studies. Summary relative risks (RRs) and 95% confidence intervals (CIs) were calculated using random effects models. Thirteen cohort studies were included. The summary relative risk was 1.21 (95% CI 1.12–1.30, I^2^ = 76%, n = 8) per 5 unit increment in BMI, 1.16 (95% CI 1.12–1.19, I^2^ = 0%, n = 5) per 10 cm increase in waist circumference, 1.06 (95% CI 1.04–1.08, I^2^ = 67%, n = 3) per 5 kg increase in weight and 1.12 (95% CI 1.06–1.18, I^2^ = 86%, n = 3) per 5 kg of weight gain. The summary RR was 1.16 (95% CI 1.03–1.31, I^2^ = 51%, n = 10) for participants with diabetes compared to participants without diabetes, and 0.93 (95% CI 0.78–1.10, I^2^ = 80%, n = 4) for high vs. low physical activity. These results suggest a positive association between adiposity and diabetes and the risk of kidney stones, but no association with physical activity.

**Electronic supplementary material:**

The online version of this article (10.1007/s10654-018-0426-4) contains supplementary material, which is available to authorized users.

## Introduction

Kidney stones are a major cause of morbidity and affects approximately 1–15% of the population around the world [1], with an incidence rate twice as high among men as compared to women [2]. The economic costs due to treatment of kidney stones in the U.S. have been estimated at 2 billion US dollar [3]. A large variation in rates of kidney stones is observed globally with a prevalence of 1–5% in Asia, 5–9% in Europe and 7–15% in North America [4]. The large variation in the rates as well as secular trend studies showing increased incidence rates in recent years [1, 5, 6] might suggest that environmental factors including diet and lifestyle may be important [7, 8]. However, improved detection of asymptomatic stones might also partly be an explanation for these trends [5]. The observation that a history of kidney stones has been associated with increased risk of kidney cancer [9], chronic kidney disease [10, 11], and more recently also with cardiovascular disease [12] might suggest that kidney stones may share some of the same risk factors as these conditions.

Several epidemiological studies have reported increased risk of kidney stones with greater body mass index (BMI, weight in kg/height in m^2^) [13–15], however, other studies found no clear association [16]. In addition, several studies also found a positive association between greater waist circumference and weight gain and risk of kidney stones [13, 16], although this was not consistently observed [16, 17]. Adiposity is related to insulin resistance and there is a growing body of evidence suggesting that insulin resistance also may play a role in the etiology of kidney stones [18]. Several [17, 19, 20], but not all studies [21–24] have reported increased risk of kidney stones among diabetes patients compared to persons without diabetes, although this has been more consistently observed among studies of women than among men [19, 24]. In contrast, there is evidence that physical activity may reduce weight gain [25] and risk of type 2 diabetes [26], and could therefore also potentially have a beneficial role in relation to risk of kidney stones, however, the available evidence is currently limited and inconsistent with one study showing a reduced risk [14], while three other studies found no significant association [27]. Although some case–control studies also support an adverse association between adiposity and diabetes and kidney stones [28–34], recall bias and selection biases can affect these studies. Therefore, to clarify the association between adiposity, diabetes and physical activity and the risk of kidney stones we conducted a systematic review and meta-analysis of published cohort studies. We particularly wanted to clarify the strength and shape of the dose–response relationship for adiposity and physical activity and potential confounding and sources of heterogeneity across studies.

## Methods

### Search strategy and inclusion criteria

We searched the Pubmed and Embase up to April 22nd 2018 for eligible studies. The search terms used are provided in the Supplementary Text. We followed standard criteria for reporting meta-analyses of observational studies [35]. In addition, we searched the reference lists of the relevant publications for further studies.

### Study selection

Cohort studies of the association between BMI, weight, weight change, waist circumference, diabetes, and physical activity and risk of kidney stones were included. Relative risk (RR) estimates (hazard ratio, risk ratio) had to be available with the 95% confidence intervals (CIs) in the publication and for the dose–response analysis, a quantitative measure of the exposure and the total number of cases and person-years or non-cases had to be available in the publication. A list of the excluded studies and exclusion reasons are found in Supplementary Table 1.

**Table 1:** Subgroup analyses of BMI and kidney stones

	BMI, per 5 kg/m^2^				
	*n*	RR (95% CI)	*I*^*2*^ (%)	*P*_h_^a^	*P*_h_^b^
All studies	8	1.21 (1.12–1.30)	76.4	< 0.0001	
Sex					
Men	3	1.24 (1.13–1.36)	0	0.64	0.72/0.96^c^
Women	4	1.22 (1.05–1.42)	88.5	< 0.0001	
Men and women	1	1.09 (0.86–1.37)			
Assessment of weight/height					
Measured	5	1.11 (1.07–1.16)	0	0.62	0.003
Self-reported	3	1.32 (1.23–1.41)	32.4	0.23	
Duration of follow-up					
< 10 years follow-up	3	1.12 (1.07–1.17)	0	0.91	0.14
≥ 10 years follow-up	5	1.27 (1.14–1.41)	78.4	0.001	
Geographic location					
Europe	0				0.20
America	4	1.26 (1.12–1.41)	86.9	< 0.0001	
Asia	4	1.12 (1.05–1.18)	0	0.45	
Number of cases					
Cases < 500	0				0.99
Cases 500 to < 1000	2	1.22 (0.91–1.66)	46.6	0.17	
Cases ≥ 1000	4	1.26 (1.12–1.41)	86.9	< 0.0001	
Study quality					
0–3	0				0.36
4–6	1	1.49 (1.01–2.21)			
7–9	7	1.20 (1.11–1.29)	78.7	< 0.0001	
Adjustment for confounders					
Age					
Yes	8	1.21 (1.12–1.30)	76.4	< 0.0001	NC
No	0				
Alcohol					
Yes	4	1.29 (1.20–1.40)	45.0	0.14	0.007
No	4	1.12 (1.07–1.16)	0	0.46	
Smoking					
Yes	4	1.12 (1.05–1.18)	0	0.45	0.20
No	4	1.26 (1.12–1.41)	86.9	< 0.0001	
Diabetes					
Yes	4	1.11 (1.07–1.15)	0	0.93	0.002
No	4	1.32 (1.25–1.41)	10.0	0.34	
Thiazide use					
Yes	3	1.32 (1.23–1.41)	32.4	0.23	0.003
No	5	1.11 (1.07–1.16)	32.4	0.23	
Fluids					
Yes	3	1.32 (1.23–1.41)	32.4	0.23	0.003
No	5	1.11 (1.07–1.16)	0	0.62	
Sodium					
Yes	4	1.29 (1.20–1.40)	45.0	0.14	0.007
No	4	1.12 (1.07–1.16)	0	0.46	
Potassium					
Yes	6	1.22 (1.11–1.33)	76.2	0.001	0.69
No	2	1.21 (0.93–1.56)	53.0	0.15	
Animal protein, protein					
Yes	5	1.23 (1.12–1.35)	80.1	< 0.0001	0.43
No	3	1.12 (1.04–1.21)	8.5	0.34	
Dietary calcium					
Yes	7	1.20 (1.11–1.29)	78.7	< 0.0001	0.36
No	1	1.49 (1.01–2.21)			
Calcium supplements					
Yes	3	1.12 (1.04–1.21)	8.5	0.34	0.003
No	5	1.30 (1.21–1.40)	32.2	0.21	
Physical activity					
Yes	5	1.11 (1.07–1.16)	0	0.62	0.003
No	3	1.32 (1.23–1.41)	32.4	0.23	

*n* denotes the number of studies

^a^P for heterogeneity within each subgroup

^b^P for heterogeneity between subgroups

^c^P for heterogeneity between men and women (excluding men/women combined)

### Data extraction

The following data were extracted from each study: The first author’s last name, publication year, country where the study was conducted, study period, sample size, number of cases/controls, exposure variable, exposure level, relative risks and 95% confidence intervals for the highest versus the lowest level of the exposure variable and variables adjusted for in the analysis. Data were extracted by DA and the extractions were checked for accuracy by YMS.

### Statistical analysis

We calculated summary RRs and 95% CIs for a 5 unit increment in BMI, 5 kg increment in weight and weight gain, 10 cm increment in waist circumference, and per 20 MET-hours of physical activity per week and a diabetes diagnosis versus no diabetes using a random effects model [36]. The average of the natural logarithm of the RRs was estimated and the RR from each study was weighted using random effects weights [36]. A two-tailed *p* < 0.05 was considered statistically significant.

The method described by Greenland and Longnecker [37] was used for the dose–response analysis and study-specific slopes (linear trends) and 95% CIs were computed from the natural logs of the RRs and CIs across categories of anthropometric measures and physical activity. The method requires that the distribution of cases and person-years or non-cases and the RRs with the variance estimates for at least three quantitative exposure categories are known. The mean BMI, waist circumference of waist-to-hip ratio level in each category was assigned to the corresponding relative risk for each study and for studies that reported the exposures in ranges we calculated the average of the upper and the lower cut-off point which was used as a midpoint. A potential nonlinear dose–response relationship between BMI, weight, weight gain and physical activity and risk of kidney stones was examined by using fractional polynomial models [38]. We determined the best fitting second order fractional polynomial regression model, defined as the one with the lowest deviance. A likelihood ratio test was used to assess the difference between the nonlinear and linear models to test for nonlinearity [38].

Subgroup and meta-regression analyses were conducted to investigate potential sources of heterogeneity including study characteristics such as duration of follow-up, sex, geographic location, study quality and adjustment for confounding factors. Heterogeneity between studies was quantitatively assessed by the Q test and I^2^ [39]. Small study effects, such as publication bias, were assessed by inspecting the funnel plots for asymmetry and with Egger’s test [40] and Begg’s test [41] with the results considered to indicate small study effects when *p* < 0.10. Sensitivity analyses excluding one study at a time were conducted to clarify whether the results were simply due to one large study or a study with an extreme result. Study quality was assessed using the Newcastle–Ottawa scale [42], which scores studies on a scale from 0 to 9 on selection, comparability and outcome assessment.

## Results

**Fig. 1:**
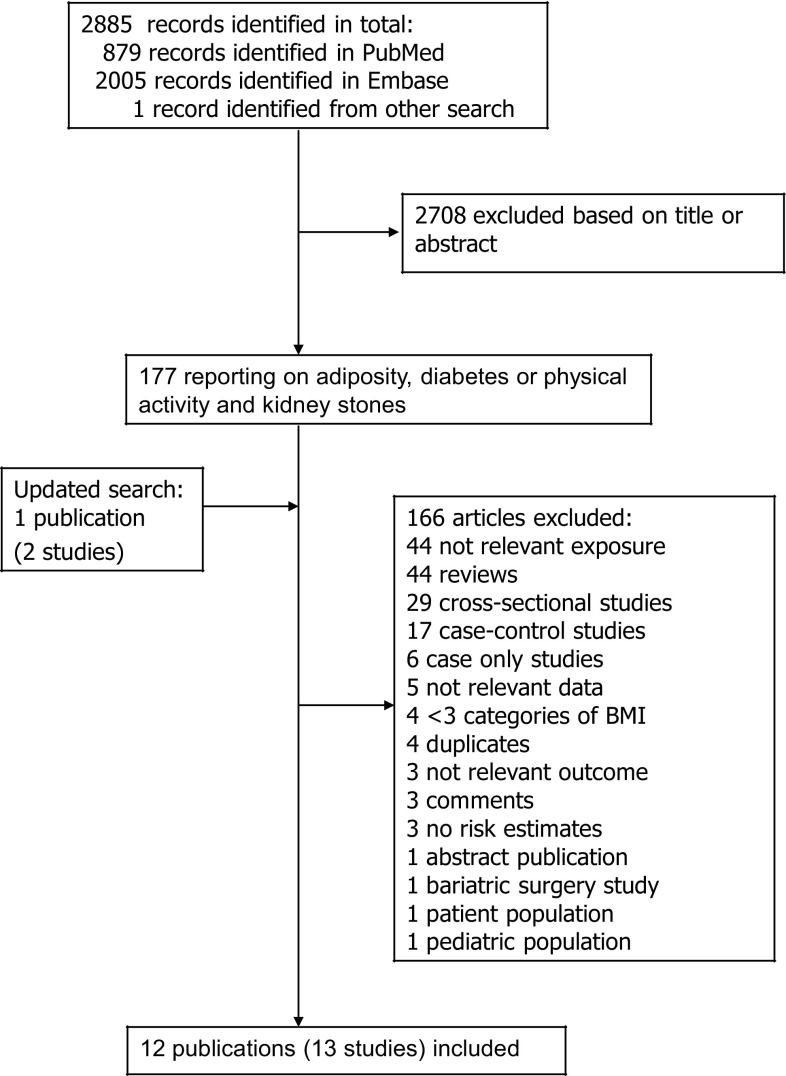
Flow-chart of study selection

We identified thirteen cohort studies (12 publications) [13–17, 19–24, 27] that could be included in the meta-analysis, including nine cohort studies (six publications) [13–17, 24] that were included in the analyses of adiposity and risk of kidney stones (Supplementary Table 2, Fig. 1), ten studies (seven publications) [17, 19–24] of diabetes and kidney stones (Supplementary Table 3, Fig. 1) and four studies (two publications) [14, 27] of physical activity and kidney stones (Supplementary Table 4, Fig. 1). Characteristics of the included studies are provided in Supplementary Table 2–4. For adiposity five studies were from the USA, and four were from Asia. Three studies were only in men, four only in women and two studies in men and women combined (Supplementary Table 2). The number of participants ranged from 4074 to 101877 and the duration of follow-up ranged from 3.2 to 20 years (Supplementary Table 2). Five studies of diabetes were from the USA, two from Europe and three from Asia (Supplementary Table 3). Two studies were in men, three in women and five in men and women combined (Supplementary Table 3). The number of participants ranged from 12,161 to 168,646 and the duration of follow-up ranged from 3.45 to 20 years (Supplementary Table 3). In the analysis of physical activity and kidney stones all the four studies were from the USA (Supplementary Table 4). Three of the studies were in women and one in men and the number of participants ranged from 44,964 to 95,618 (Supplementary Table 4). Mean (median) study quality scores for the studies of BMI were 7.3 (7.0), for the studies of diabetes mellitus were 7.2 (7.0), and for the studies of physical activity were 7.0 (7.0).

**Table 2:** Subgroup analyses of diabetes and kidney stones

	Diabetes				
	*n*	RR (95% CI)	*I*^*2*^ (%)	*P*_h_^a^	*P*_h_^b^
All studies	10	1.16 (1.03–1.31)	50.5	0.03	
Sex					
Men	2	0.91 (0.75–1.10)	0	0.34	0.24/0.09^c^
Women	3	1.29 (1.08–1.55)	32.5	0.23	
Men and women	5	1.19 (1.02–1.38)	33.6	0.20	
Duration of follow-up					
< 10 years follow-up	5	1.12 (1.01–1.24)	0	0.53	0.41
≥ 10 years follow-up	5	1.25 (0.98–1.61)	71.2	0.008	
Geographic location					
Europe	2	1.23 (1.06–1.44)	0	0.71	0.15
America	5	1.25 (0.98–1.59)	71.9	0.007	
Asia	3	1.04 (0.90–1.20)	0	0.80	
Number of cases					
Cases < 500	0				0.87
Cases 500 to < 1000	4	1.18 (0.94–1.48)	43.8	0.15	
Cases ≥ 1000	6	1.15 (0.99–1.35)	60.6	0.03	
Study quality					
0–3	0				NC
4–6	2	1.04 (0.87–1.24)	0	0.50	
7–9	8	1.20 (1.03–1.38)	56.0	0.03	
Adjustment for confounders					
Age					
Yes	10	1.16 (1.03–1.31)	50.5	0.03	NC
No	0				
Alcohol					
Yes	3	1.19 (0.84–1.69)	79.7	0.007	0.80
No	7	1.14 (1.02–1.27)	24.4	0.24	
Smoking					
Yes	3	1.06 (0.92–1.22)	0	0.74	0.38
No	7	1.22 (1.02–1.45)	60.8	0.02	
BMI, adiposity					
Yes	5	1.14 (1.03–1.26)	0	0.61	0.54
No	5	1.24 (0.96–1.59)	73.5	0.005	
Thiazide use					
Yes	3	1.19 (0.84–1.69)	79.7	0.007	0.80
No	7	1.14 (1.02–1.27)	24.4	0.24	
Fluids					
Yes	3	1.19 (0.84–1.69)	79.7	0.007	0.80
No	7	1.14 (1.02–1.27)	24.4	0.24	
Sodium					
Yes	3	1.19 (0.84–1.69)	79.7	0.007	0.80
No	7	1.14 (1.02–1.27)	24.4	0.24	
Potassium					
Yes	5	1.13 (0.92–1.38)	66.4	0.02	0.69
No	5	1.19 (1.02–1.38)	33.6	0.20	
Animal protein					
Yes	5	1.13 (0.92–1.38)	66.4	0.02	0.69
No	5	1.19 (1.02–1.38)	33.6	0.20	
Dietary calcium					
Yes	5	1.13 (0.92–1.38)	66.4	0.02	0.69
No	5	1.19 (1.02–1.38)	33.6	0.20	
Calcium supplements					
Yes	5	1.13 (0.92–1.38)	66.4	0.02	0.69
No	5	1.19 (1.02–1.38)	33.6	0.20	
Physical activity					
Yes	2	1.04 (0.87–1.24)	0	0.50	0.43
No	8	1.20 (1.03–1.38)	56.0	0.03	

*n* denotes the number of studies

^a^P for heterogeneity within each subgroup

^b^P for heterogeneity between subgroups

^c^P for heterogeneity between men and women (excluding men/women combined)

### Body mass index

**Fig. 2:**
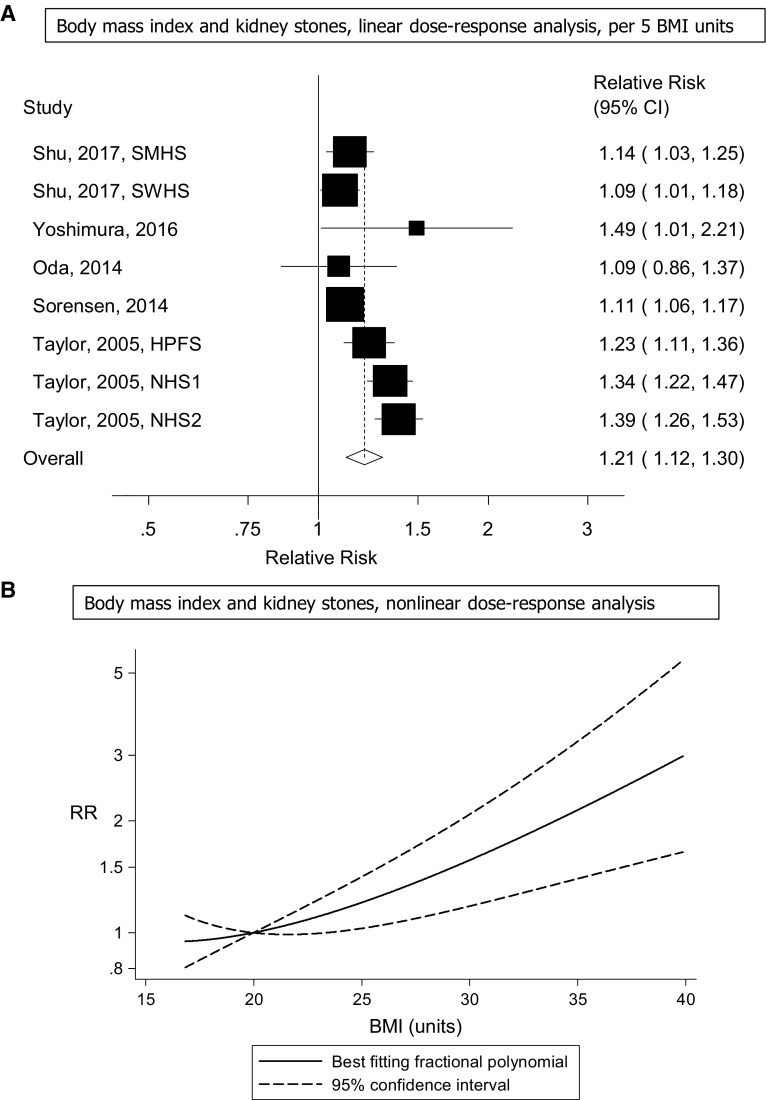
Body mass index and risk of kidney stones

Eight cohort studies (five publications) [13–16, 24] were included in the dose–response analysis of BMI and risk of kidney stones and included a total of 10,368 cases among 458,868 participants. The summary RR for a 5 unit increment in BMI was 1.21 (95% CI 1.12–1.30, I^2^ = 76% [95% CI 53–88%], p_heterogeneity_ < 0.0001) (Fig. 2A). In sensitivity analyses excluding the most influential studies, the summary RR ranged from 1.17 (95% CI 1.10–1.25) when excluding the Nurses’ Health Study 2 [13] to 1.23 (95% CI 1.13–1.34) when excluding the Shanghai Women’s Health Study [24] (Supplementary Figure 1). The heterogeneity was reduced when excluding the Nurses’ Health Study 1 and 2 (which both reported stronger associations than most of the remaining studies) [13], but the association remained significant, summary RR = 1.13 (95% CI 1.09–1.18, I^2^ = 12%, p_heterogeneity_ = 0.34). There was no indication of publication bias with Egger’s test, *p* = 0.28, or with Begg’s test, *p* = 0.17 (Supplementary Figure 2). There was evidence of a nonlinear association between BMI and risk of kidney stones, p_nonlinearity_ < 0.0001, and the curve was flat between 20 and 22, and increased at BMI values above this range (Fig. 2B, Supplementary Table 5).

### Waist circumference

**Fig. 3:**
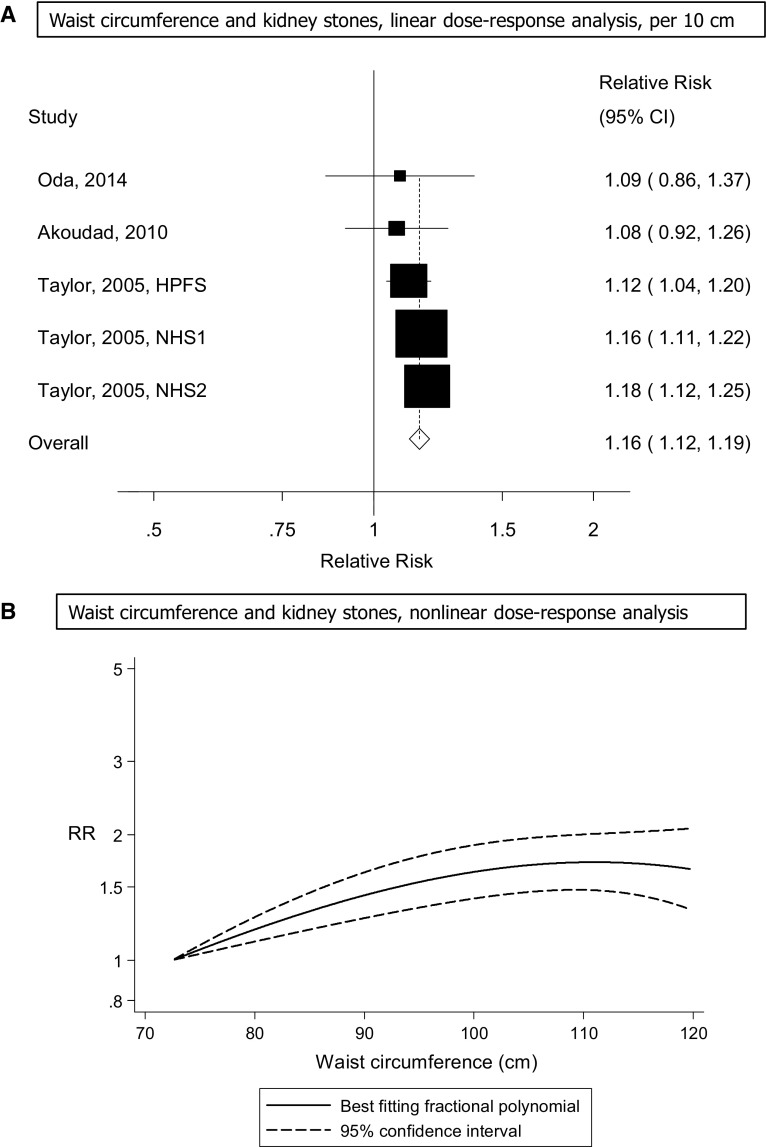
Waist circumference and risk of kidney stones

Five cohort studies (three publications) [13, 16, 17] were included in the analysis of waist circumference and risk of kidney stones and included 4282 cases among 255,510 participants. The summary RR for a 10 cm increase in waist circumference was 1.16 (95% CI 1.12–1.19, I^2^ = 0% [95% CI 0–79%], p_heterogeneity_ = 0.59) (Fig. 3A). The summary RR ranged from 1.14 (95% CI 1.10–1.19) when the Nurses’ Health Study 2 [13] was excluded to 1.17 (95% CI 1.12–1.21) when the Health Professionals Follow-up Study [13] was excluded (Supplementary Figure 3). There was some indication of a nonlinear association between waist circumference and the risk of kidney stones with a slight flattening of the curve at higher levels (around 105–110 cm), p_nonlinearity_ = 0.03 (Fig. 3B, Supplementary Table 6).

### Weight

**Fig. 4:**
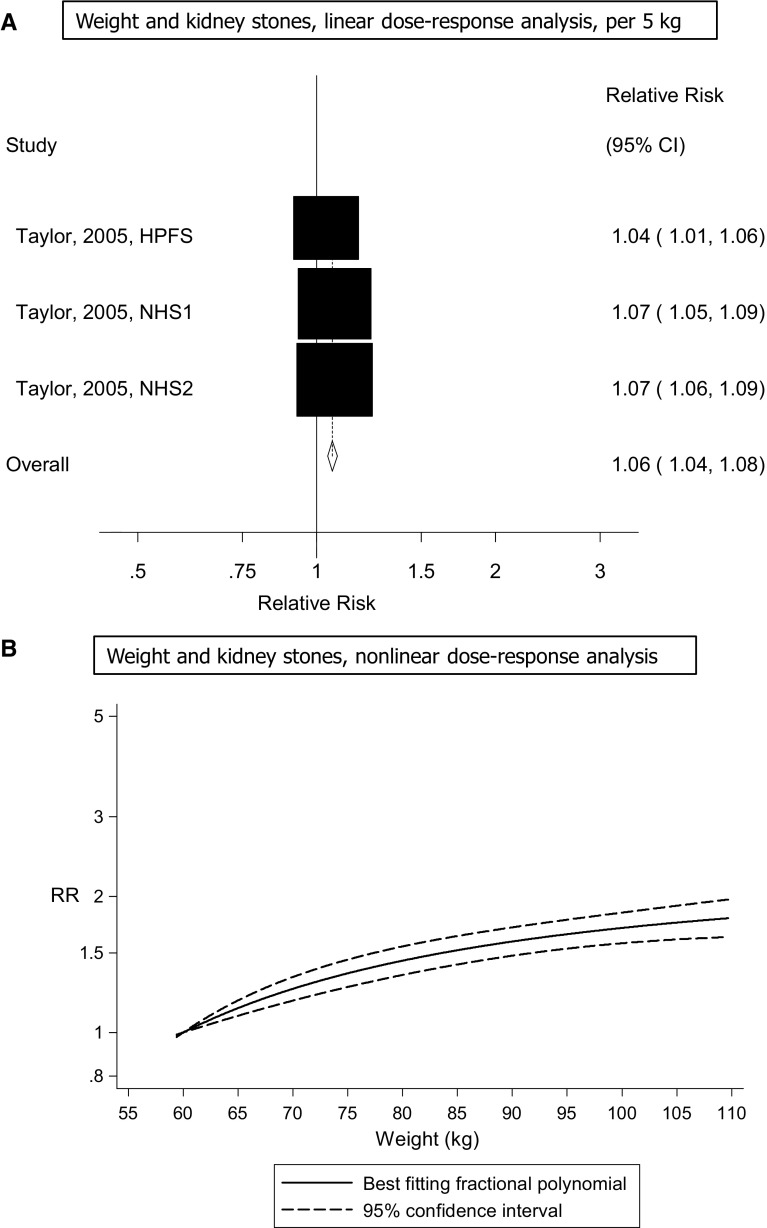
Weight and risk of kidney stones

Three cohort studies (one publication) [13] were included in the analyses of weight and the risk of kidney stones and included 4827 cases among 241,623 participants. The summary RR per 5 kg increment in weight was 1.06 (95% CI 1.04–1.08, I^2^ = 67% [95% CI 0–90%], p_heterogeneity_ < 0.0001) (Fig. 4A). The summary RR ranged from 1.06 (95% CI 1.02–1.09) when the Nurses’ Health Study 2 [13] was excluded to 1.07 (95% CI 1.06–1.09) when the Health Professional’s Follow-up Study [13] was excluded and there was no heterogeneity when the latter study was excluded from the analysis, I^2^ = 0%, p_heterogeneity_ = 0.99. There was evidence of a nonlinear association between weight and risk of kidney stones risk, p_nonlinearity_ < 0.0001 (Fig. 4B, Supplementary Table 7).

### Weight gain

**Fig. 5:**
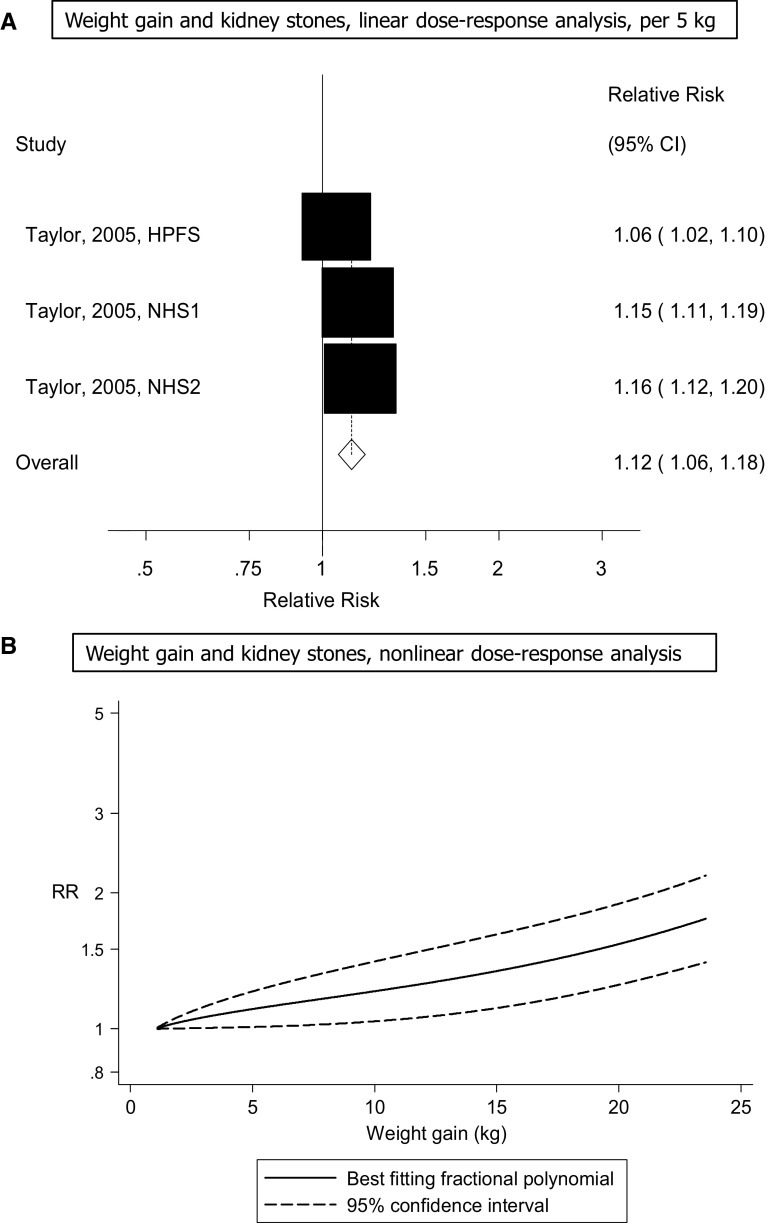
Weight gain and risk of kidney stones

Three cohort studies (one publication) [13] were included in the analyses of weight gain (change in recalled body weight between age 21 in men and age 18 in women and baseline of the studies) and the risk of kidney stones and included 4575 cases among 241,623 participants. The summary RR per 5 kg increment in weight gain was 1.12 (95% CI 1.06–1.18, I^2^ = 86% [95% CI 59–95%], p_heterogeneity_ < 0.0001) (Fig. 5A). The summary RR ranged from 1.10 (95% CI 1.02–1.20) when the Nurses’ Health Study [13] was excluded to 1.15 (95% CI 1.13–1.18) when the Health Professional’s Follow-up Study [13] was excluded and there was no heterogeneity when the latter study was excluded from the analysis, I^2^ = 0%, p_heterogeneity_ = 0.68. There was no evidence of a nonlinear association between weight gain and risk of kidney stones, p_nonlinearity_ = 0.12 (Fig. 5B, Supplementary Table 8).

### Diabetes

**Fig. 6:**
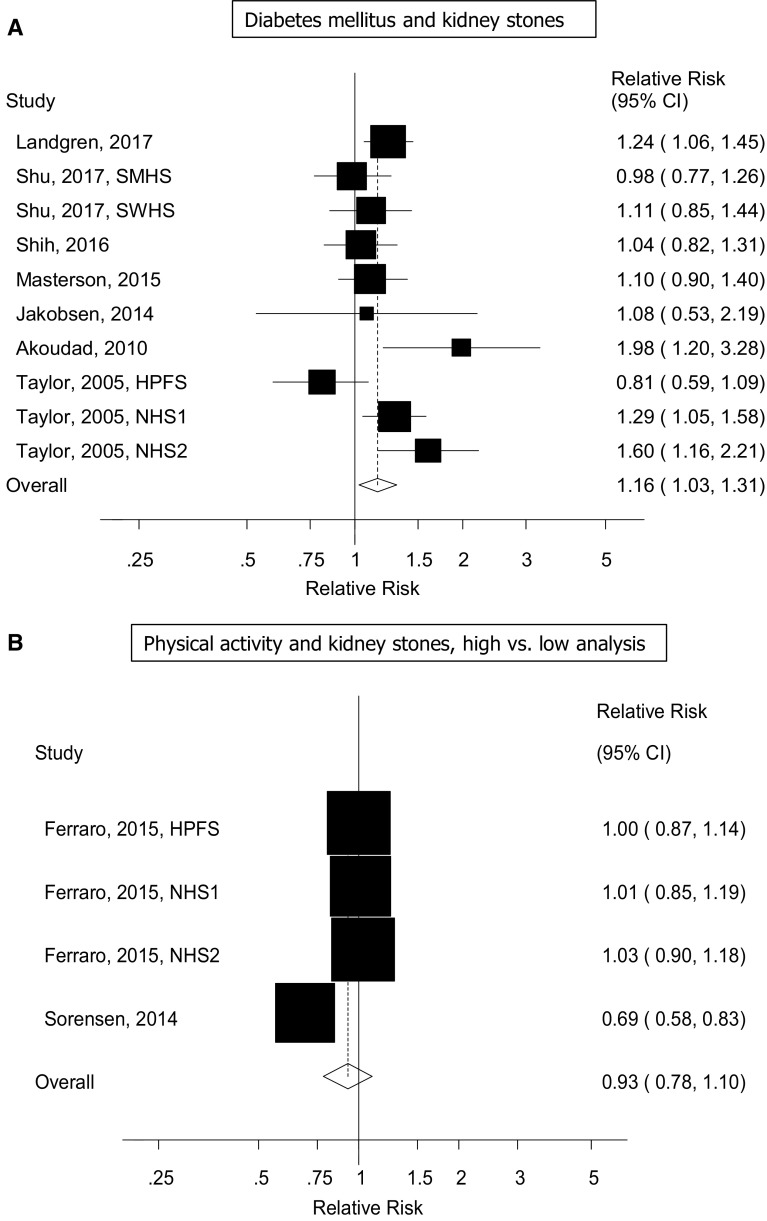
Diabetes mellitus and physical activity and risk of kidney stones

Ten cohort studies (seven publications) [17, 19–24] were included in the analysis of diabetes and the risk of kidney stones and included 13,475 cases and 666,715 participants. The summary RR for diabetes patients compared to persons without diabetes was 1.16 (95% CI 1.03–1.31, I^2^ = 51% [95% CI 0–76%], p_heterogeneity_ = 0.03) (Fig. 6A). The summary RR ranged from 1.12 (95% CI 1.00–1.26) when excluding the Nurses’ Health Study 2 [19] to 1.19 (95% CI 1.07–1.33) when excluding the Health Professionals Follow-up Study [19] (Supplementary Figure 4). There was no evidence of publication bias with Egger’s test, *p* = 0.86, or with Begg’s test, *p* = 0.84 (Supplementary Figure 5).

### Physical activity

**Fig. 7:**
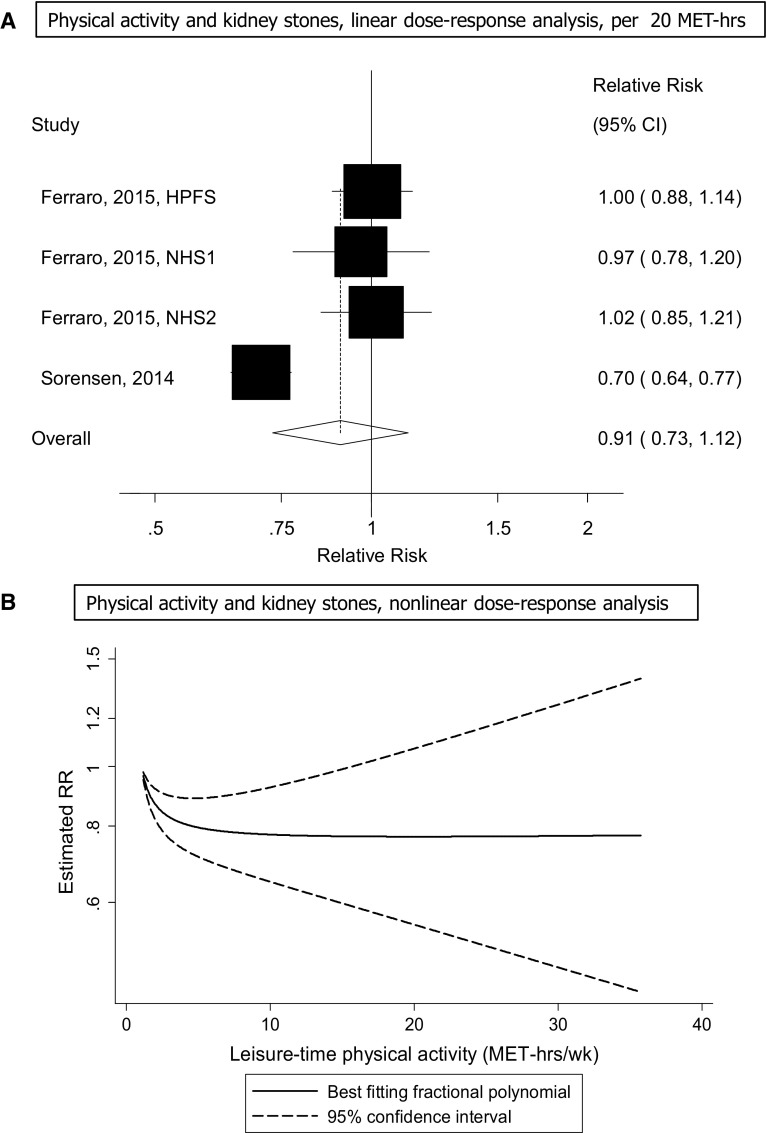
Physical activity and risk of kidney stones

Four cohort studies (two publications) [14, 27] were included in the analysis of physical activity and the risk of kidney stones and included 7747 cases and 367,319 participants. The summary RR for the highest vs. the lowest level of physical activity was 0.93 (95% CI 0.78–1.10, I^2^ = 80% [95% CI 46–92%], p_heterogeneity_ < 0.0001) (Fig. 7). The summary RR per 20 MET-hours per week was 0.96 (95% CI 0.88–1.05, I^2^ = 88% [73–95%], p_heterogeneity_ < 0.0001) (Fig. 7a). The test for nonlinearity was significant, p_nonlinearity_ < 0.0001, and the association was significant at 1–2 up to 15 MET-hours per week of activity and the confidence intervals became much wider at higher levels of activity (Fig. 7b, Supplementary Table 8).

### Subgroup, sensitivity analyses, and study quality

The positive association between BMI and kidney stones persisted in most subgroup analyses defined by sex, assessment of anthropometric measures, duration of follow-up, number of cases and adjustment for confounding factors. There was heterogeneity between several of the subgroup analyses including measured versus self-reported weight/height data (p_heterogeneity_ = 0.003) with stronger associations among studies with self-reported data vs. those with measured data. There was also heterogeneity by adjustment for alcohol (p_heterogeneity_ = 0.007), adjustment for diabetes (p_heterogeneity_ = 0.002), thiazide use (p_heterogeneity_ = 0.003), fluids (p_heterogeneity_ = 0.003), sodium (p_heterogeneity_ = 0.007), calcium supplements (p_heterogeneity_ = 0.003), and physical activity (p_heterogeneity_ = 0.003) with weaker associations among studies with adjustment for diabetes, calcium supplements and physical activity versus studies without such adjustment and stronger associations among studies with adjustment for alcohol, thiazide use, fluids, and sodium than among studies without such adjustment (Table 1). The positive association between diabetes mellitus and kidney stones was not significant in every subgroup analysis, however, there was no evidence of between-subgroup heterogeneity (Table 2). The association was positive among two studies of women, but not in one study of men (Table 2).

There was no heterogeneity when results were stratified by study quality (Tables 1, 2).

## Discussion

To our knowledge this is the first dose–response meta-analysis of body fatness, diabetes and physical activity in relation to risk of kidney stones risk and we found a 21% increase in the relative risk per 5 units increase in BMI, 16% increase in relative risk per 10 cm increase in waist circumference, and 6 and 12% increases in the relative risk per 5 kg increment in weight and weight gain, and a 16% increase in the relative risk of kidney stones among diabetes patients compared to persons without diabetes. There was no significant association between physical activity and kidney stones in the high vs. low and linear dose–response analyses, but some suggestion of a possible nonlinear association with moderate levels of physical activity showing an inverse association.

Our meta-analysis has some limitations that need to be mentioned. The main limitation is the low number of cohort studies available reporting on waist circumference, weight, weight changes, and physical activity which limited our possibility to conduct subgroup analyses and sensitivity analyses. No studies reported on waist-to-hip ratio, waist-to-height ratio or adiposity in adolescence or early adulthood and risk of kidney stones. We cannot entirely exclude the potential for residual confounding (for example by smoking) having affected the findings, however, the association between BMI and kidney stones persisted in several subgroup analyses with adjustment for a number of confounding factors and there was no between subgroup heterogeneity. Nevertheless, confounding by risk factors that were not included in the statistical analyses or by risk factors that are not yet known is still possible. Measurement errors could have influenced the findings. When the analysis was stratified by whether the anthropometric factors were measured or self-reported the summary estimates were stronger among the studies with self-reported anthropometric measures compared to those with measured data, which might suggest overestimation of the association when using self-reported anthropometrics. However, it is also possible that this difference is due to differences in other study characteristics between studies. Ideally one should compare analyses of measured and self-reported data within the same study. As a meta-analysis of published literature it is possible that publication bias may have affected our findings, but we did not find evidence of such bias in the analysis of BMI or diabetes with the statistical tests used, however, the limited number of studies in the remaining analyses did not permit formal testing. There was high heterogeneity in the main analyses for BMI, weight and weight gain, however, this appeared to be explained to a large extent by one or two studies [13] in each analysis and when excluded the summary estimates remained significant. Within subgroups there was lower heterogeneity among studies in men, among studies with either measured or self-reported weight and height, among the Asian studies, and among studies with adjustment for alcohol, smoking, diabetes, thiazide use, fluids, sodium, calcium supplements, and physical activity. In subgroup and meta-regression analyses the positive association between BMI and risk of kidney stones persisted in most subgroup defined by sex, exposure assessment, duration of follow-up, number of cases and adjustment for several important confounding factors. There was significant heterogeneity between several of the subgroups, with weaker associations among studies with adjustment for diabetes, calcium supplement use and physical activity compared to studies with such adjustment, but stronger associations among studies with adjustment for intake of alcohol, thiazide use, fluids and sodium compared to studies without such adjustment. However, because of the limited number of studies and because some studies tended to cluster together in several of the subgroup analyses it is not clear if those study characteristics or some other correlated study characteristics truly caused some of the heterogeneity. Although the test for heterogeneity was not significant when studies were stratified by geographic location, the association between BMI and risk of kidney stones appeared to be slightly stronger in the American than in the Asian studies and a positive association between diabetes mellitus and kidney stones was restricted to the European and American studies and not observed in the Asian studies. This might at least partly be due to higher levels of adiposity and higher rates of type 2 diabetes in the European and American studies compared to the Asian studies [43, 44].

Weight, weight gain and waist circumference are highly correlated with BMI and because none of the studies made further mutual adjustments between adiposity variables (for example between BMI and waist circumference) we are not able to conclude whether general or abdominal adiposity is more or equally important in increasing the risk of kidney stones and this is something that could be further clarified in future studies.

Strengths of our analysis include the prospective design of the studies which therefore avoids recall bias, which can affect retrospective case–control studies, and limits the possibility that selection bias explains the results. The meta-analysis included > 10,300 cases among > 458,000 participants in the BMI analysis and > 13,400 cases and > 666,000 participants in the diabetes analysis, providing sufficient statistical power to detect even modest associations. To increase comparability between studies we conducted linear and nonlinear dose–response analyses. The results persisted in a number of subgroup and sensitivity analyses, suggesting that the findings are not likely to be completely explained by confounding. The results were robust to the influence of single studies, and the included studies had a relatively high study quality.

Several potential mechanisms could explain an association between body fatness and increased risk of kidney stones. Obesity has been associated with high serum uric acid and gout [45, 46], gallstones [47], insulin resistance and type 2 diabetes [48], which again is associated with increased risk of kidney stones [17, 19, 49–51]. In subgroup analyses there was a weaker association among studies that adjusted for diabetes than among studies that did not adjust for diabetes in the analysis of BMI (RR = 1.11 vs. 1.32). This might suggest that part of the association between BMI and kidney stones may be mediated by type 2 diabetes and that the current summary estimate might be a conservative estimate of the risk of kidney stones associated with adiposity. However, within study comparisons with analyses with and without adjustment for diabetes are needed to draw firm conclusions. It has been shown that insulin suppresses calcium reabsorption by acting on the renal tubules [18], and that insulin promotes the urinary and fractional excretion of calcium [52]. Other studies have reported that obesity increases urinary oxalate excretion [46] and reduces urinary citrate excretion [53]. A rat model found that a weight loss intervention reduced the risk of kidney stone formation and the intervention group had higher urinary pH and higher excretion of urinary citrate than control rats [54].

The current findings have important public health implications in light of the current epidemics of overweight and obesity [43] and diabetes [44] globally and suggest that if current trends in rates of obesity and diabetes continue an increased incidence of kidney stones could result. Since a history of kidney stones is a risk factor for kidney cancer [9] and chronic kidney disease [11] and is potentially also related to increased cardiovascular disease risk [12] further complications might also be a consequence.

Our findings confirm that body fatness is an important risk factor for kidney stones and there was a 1.4-fold increase in risk among overweight and 2–3 fold increase in risk among obese and severely obese participants, respectively, while the lowest risk observed in the BMI range between 20 and 22. In addition, both waist circumference, weight and weight gain was associated with an increased risk, although additional studies are needed because of the limited data on these adiposity measures. A diagnosis of diabetes was associated with a 16% increase in the relative risk compared to persons without diabetes. No association was observed between physical activity and kidney stones, however, the possibility of a nonlinear association with reductions at low-to-moderate levels of activity cannot be excluded, but further studies are needed.

Contribution: DA conceived of and designed the study. DA conducted the literature search and analyses and wrote the first draft of the paper. DA and MSY conducted the literature screening. All authors interpreted the data, contributed to the draft of the paper, revised the subsequent drafts for important intellectual content, read and approved the final manuscript. D. Aune takes primary responsibility for the integrity of the data and the accuracy of the data analysis.

## Electronic supplementary material

Below is the link to the electronic supplementary material.

Supplementary material 1 (PDF 340 kb)

## References

[1] Romero V, Akpinar H, Assimos DG (2010). Kidney stones: a global picture of prevalence, incidence, and associated risk factors. Rev Urol.

[2] Johnson CM, Wilson DM, O’Fallon WM, Malek RS, Kurland LT (1979). Renal stone epidemiology: a 25-year study in Rochester, Minnesota. Kidney Int.

[3] Lingeman JE, Saywell RM, Woods JR, Newman DM (1986). Cost analysis of extracorporeal shock wave lithotripsy relative to other surgical and nonsurgical treatment alternatives for urolithiasis. Med Care.

[4] Ramello A, Vitale C, Marangella M (2000). Epidemiology of nephrolithiasis. J Nephrol.

[5] Edvardsson VO, Indridason OS, Haraldsson G, Kjartansson O, Palsson R (2013). Temporal trends in the incidence of kidney stone disease. Kidney Int.

[6] Stamatelou KK, Francis ME, Jones CA, Nyberg LM, Curhan GC (2003). Time trends in reported prevalence of kidney stones in the United States: 1976–1994. Kidney Int.

[7] Turney BW, Appleby PN, Reynard JM, Noble JG, Key TJ, Allen NE (2014). Diet and risk of kidney stones in the Oxford cohort of the European Prospective Investigation into Cancer and Nutrition (EPIC). Eur J Epidemiol.

[8] Ahmed MH, Ahmed HT, Khalil AA (2012). Renal stone disease and obesity: what is important for urologists and nephrologists?. Ren Fail.

[9] Cheungpasitporn W, Thongprayoon C, O’Corragain OA (2015). The risk of kidney cancer in patients with kidney stones: a systematic review and meta-analysis. QJM.

[10] Shang W, Li L, Ren Y (2017). History of kidney stones and risk of chronic kidney disease: a meta-analysis. PeerJ.

[11] Hippisley-Cox J, Coupland C (2010). Predicting the risk of chronic kidney disease in men and women in England and Wales: prospective derivation and external validation of the QKidney Scores. BMC Fam Pract.

[12] Ferraro PM, Taylor EN, Eisner BH (2013). History of kidney stones and the risk of coronary heart disease. JAMA.

[13] Taylor EN, Stampfer MJ, Curhan GC (2005). Obesity, weight gain, and the risk of kidney stones. JAMA.

[14] Sorensen MD, Chi T, Shara NM (2014). Activity, energy intake, obesity, and the risk of incident kidney stones in postmenopausal women: a report from the Women’s Health Initiative. J Am Soc Nephrol.

[15] Yoshimura E, Sawada SS, Lee IM (2016). Body mass index and kidney stones: a cohort study of Japanese men. J Epidemiol.

[16] Oda E (2014). Overweight and high-sensitivity C-reactive protein are weakly associated with kidney stone formation in Japanese men. Int J Urol.

[17] Akoudad S, Szklo M, McAdams MA (2010). Correlates of kidney stone disease differ by race in a multi-ethnic middle-aged population: the ARIC study. Prev Med.

[18] Ando R, Suzuki S, Nagaya T (2011). Impact of insulin resistance, insulin and adiponectin on kidney stones in the Japanese population. Int J Urol.

[19] Taylor EN, Stampfer MJ, Curhan GC (2005). Diabetes mellitus and the risk of nephrolithiasis. Kidney Int.

[20] Landgren AJ, Jacobsson LTH, Lindstrom U (2017). Incidence of and risk factors for nephrolithiasis in patients with gout and the general population, a cohort study. Arthritis Res Ther.

[21] Jakobsen AK, Jacobsson LT, Patschan O, Askling J, Kristensen LE (2014). Is nephrolithiasis an unrecognized extra-articular manifestation in ankylosing spondylitis? A prospective population-based Swedish national cohort study with matched general population comparator subjects. PLoS ONE.

[22] Masterson JH, Woo JR, Chang DC (2015). Dyslipidemia is associated with an increased risk of nephrolithiasis. Urolithiasis.

[23] Shih MT, Tang SH, Cha TL, Wu ST, Chiang JH, Chen WC (2016). The risk of nephrolithiasis among patients with ankylosing spondylitis: a population-based cohort study. Arch Rheumatol.

[24] Shu X, Cai H, Xiang YB (2017). Nephrolithiasis among middle aged and elderly urban Chinese: a report from prospective cohort studies in Shanghai. J Endourol.

[25] Mozaffarian D, Hao T, Rimm EB, Willett WC, Hu FB (2011). Changes in diet and lifestyle and long-term weight gain in women and men. N Engl J Med.

[26] Aune D, Norat T, Leitzmann M, Tonstad S, Vatten LJ (2015). Physical activity and the risk of type 2 diabetes: a systematic review and dose-response meta-analysis. Eur J Epidemiol.

[27] Ferraro PM, Curhan GC, Sorensen MD, Gambaro G, Taylor EN (2015). Physical activity, energy intake and the risk of incident kidney stones. J Urol.

[28] Wu CC, Hung SH, Lin HC, Lee CZ, Lee HC, Chung SD (2016). Sialolithiasis is associated with nephrolithiasis: a case-control study. Acta Otolaryngol.

[29] Zhao A, Dai M, Chen YJ, Chang HE, Liu AP, Wang PY (2015). Risk factors associated with nephrolithiasis: a case-control study in China. Asia Pac J Public Health.

[30] Akarken I, Tarhan H, Ekin RG (2015). Visceral obesity: a new risk factor for stone disease. Can Urol Assoc J.

[31] Nerli R, Jali M, Guntaka AK, Patne P, Patil S, Hiremath MB (2015). Type 2 diabetes mellitus and renal stones. Adv Biomed Res.

[32] Milicevic S, Bijelic R, Krivokuca V, Bojic M, Popovic-Pejicic S, Bojanic N (2013). Correlation of the body mass index and calcium nephrolithiasis in adult population. Med Arch.

[33] Lieske JC, de la Vega LS, Gettman MT (2006). Diabetes mellitus and the risk of urinary tract stones: a population-based case-control study. Am J Kidney Dis.

[34] Hamano S, Nakatsu H, Suzuki N, Tomioka S, Tanaka M, Murakami S (2005). Kidney stone disease and risk factors for coronary heart disease. Int J Urol.

[35] Stroup DF, Berlin JA, Morton SC (2000). Meta-analysis of observational studies in epidemiology: a proposal for reporting. Meta-analysis Of Observational Studies in Epidemiology (MOOSE) group. JAMA.

[36] DerSimonian R, Laird N (1986). Meta-analysis in clinical trials. Control Clin Trials.

[37] Greenland S, Longnecker MP (1992). Methods for trend estimation from summarized dose-response data, with applications to meta-analysis. Am J Epidemiol.

[38] Bagnardi V, Zambon A, Quatto P, Corrao G (2004). Flexible meta-regression functions for modeling aggregate dose-response data, with an application to alcohol and mortality. Am J Epidemiol.

[39] Higgins JP, Thompson SG (2002). Quantifying heterogeneity in a meta-analysis. Stat Med.

[40] Egger M, Davey SG, Schneider M, Minder C (1997). Bias in meta-analysis detected by a simple, graphical test. BMJ.

[41] Begg CB, Mazumdar M (1994). Operating characteristics of a rank correlation test for publication bias. Biometrics.

[42] Wells G, Shea B, O’Connell D. et al. The Newcastle–Ottawa Scale (NOS) for assessing the quality of nonrandomised studies in meta-analyses. http://www.ohri.ca/programs/clinical_epidemiology/oxford.asp. Accessed 29 Jul 2018, 2013.

[43] NCD Risk Factor Collaboration (NCD-RisC) (2017). Worldwide trends in body-mass index, underweight, overweight, and obesity from 1975 to 2016: a pooled analysis of 2416 population-based measurement studies in 128.9 million children, adolescents, and adults. Lancet.

[44] NCD Risk Factor Collaboration (NCD-RisC) (2016). Worldwide trends in diabetes since 1980: a pooled analysis of 751 population-based studies with 4.4 million participants. Lancet.

[45] Aune D, Norat T, Vatten LJ (2014). Body mass index and the risk of gout: a systematic review and dose-response meta-analysis of prospective studies. Eur J Nutr.

[46] Taylor EN, Curhan GC (2006). Body size and 24-hour urine composition. Am J Kidney Dis.

[47] Aune D, Norat T, Vatten LJ (2015). Body mass index, abdominal fatness and the risk of gallbladder disease. Eur J Epidemiol.

[48] Abdullah A, Peeters A, de Court M, Stoelwinder J (2010). The magnitude of association between overweight and obesity and the risk of diabetes: a meta-analysis of prospective cohort studies. Diabetes Res Clin Pract.

[49] Kramer HJ, Choi HK, Atkinson K, Stampfer M, Curhan GC (2003). The association between gout and nephrolithiasis in men: the Health Professionals’ Follow-Up Study. Kidney Int.

[50] Kim S, Chang Y, Yun KE (2017). Development of nephrolithiasis in asymptomatic hyperuricemia: a cohort study. Am J Kidney Dis.

[51] Taylor EN, Chan AT, Giovannucci EL, Curhan GC (2011). Cholelithiasis and the risk of nephrolithiasis. J Urol.

[52] DeFronzo RA, Cooke CR, Andres R, Faloona GR, Davis PJ (1975). The effect of insulin on renal handling of sodium, potassium, calcium, and phosphate in man. J Clin Invest.

[53] Mandel EI, Taylor EN, Curhan GC (2013). Dietary and lifestyle factors and medical conditions associated with urinary citrate excretion. Clin J Am Soc Nephrol.

[54] Sasaki Y, Kohjimoto Y, Iba A, Matsumura N, Hara I (2015). Weight loss intervention reduces the risk of kidney stone formation in a rat model of metabolic syndrome. Int J Urol.

