# Supplementary material for: Body fatness, diabetes, physical activity and risk of kidney stones: a systematic review and meta-analysis of cohort studies
Source: Eur J Epidemiol. 2018 Jul 31;33(11):1033–47. doi: 10.1007/s10654-018-0426-4 (PMC6208979; doi:10.1007/s10654-018-0426-4)

Supplementary text. PubMed search terms

("physical activity" OR physical activity[MeSH] OR exercise OR exercise[MeSH] OR sports OR sports[MeSH] OR walking OR walking[MeSH] OR biking OR bicycling OR bicycling[MeSH] OR running OR running[MeSH] OR fitness OR "exercise test" OR exercise test[MeSH] OR inactivity OR sedentary OR diabetes OR diabetes[MeSH] OR glucose OR glucose[MeSH] OR "medical history" OR medical history[MeSH] OR “body mass index” OR body mass index[MeSH] OR BMI OR overweight OR overweight[MeSH] OR obesity OR obesity[MeSH] OR anthropometry OR anthropometry[MeSH] OR fatness OR “body fatness” OR “abdominal fatness” OR “abdominal obesity” OR abdominal obesity[MeSH] OR “waist circumference” OR "hip circumference" OR “waist-to-hip ratio” OR waist-to-hip ratio[MeSH] OR adiposity OR adiposity[MeSH] OR "weight gain" OR weight gain[MeSH] OR "weight change" OR weight change[MeSH] OR “weight loss” OR weight loss[MeSH])

AND

("kidney stones" OR kidney stones[MeSH] OR "nephrolithiasis" OR nephrolithiasis[MeSH])

AND

("case-control" OR cohort OR prospective OR longitudinal OR retrospective OR "follow-up" OR "cross-sectional" OR "hazard ratio" OR "hazard ratios" OR "relative risk" OR "relative risks" OR "incidence rate ratio" OR "incidence rate ratios" OR "odds ratio" OR odds ratios OR incidence)

Embase search terms

((body mass index or BMI or overweight or obesity or anthropometry or fatness or body fatness or abdominal fatness or abdominal obesity or waist circumference or hip circumference or waist-to-hip ratio or adiposity or weight gain or weight change or weight loss).ab,ti or body mass index/ or BMI/ or overweight/ or obesity/ or anthropometry/ or fatness/ or body fatness/ or abdominal fatness/ or abdominal obesity/ or waist circumference/ or hip circumference/ or waist-to-hip ratio/ or adiposity/ or weight gain/ or weight change/ or weight loss/ or (diabetes or glucose or medical history).ab,ti or diabetes/ or glucose/ or medical history/ or (physical activity or exercise or sports or walking or biking or bicycling or running or fitness or exercise test or inactivity or sedentary).ab,ti or physical activity/ or exercise/ or sports/ or walking/ or biking/ or bicycling/ or running/ or fitness/ or exercise test/ or inactivity/ or sedentary/)

and

((kidney stones or nephrolithiasis).ab,ti. or kidney stones/ or nephrolithiasis/)

and

(case-control or cohort or prospective or longitudinal or retrospective or follow-up or cross-sectional or hazard ratio or hazard ratios or relative risk or relative risks or incidence rate ratio or incidence rate ratios or odds ratio or odds ratios or incidence).ab,ti.

Supplementary Table 1. List of excluded studies and reason for exclusion

Exclusion reason	Reference number
<3 categories of exposure (BMI)	(1-4)
Abstract only publication	(5)
Bariatric surgery study	(6)
Case-control study	(7-23)
Case only study	(24-29)
Comment	(30-32)
Cross-sectional study	(33-61)
Duplicates	(62-65)
No risk estimates	(66-68)
Not relevant data	(69-73)
Not relevant exposure	(74-117)
Not relevant outcome	(118-120)
Patient populations	(121)
Pediatric population	(122)
Review	(123-166)

Reference List

1. Chang IH, Lee YT, Lee DM et al. Metabolic syndrome, urine pH, and time-dependent risk of nephrolithiasis in Korean men without hypertension and diabetes. Urology 2011;78:753-8.

2. Jung HS, Chang IH, Kim KD et al. Possible Relationship between Metabolic Syndrome Traits and Nephrolithiasis: Incidence for 15 Years According to Gender. Korean J Urol 2011;52:548-53.

3. Turney BW, Appleby PN, Reynard JM, Noble JG, Key TJ, Allen NE. Diet and risk of kidney stones in the Oxford cohort of the European Prospective Investigation into Cancer and Nutrition (EPIC). Eur J Epidemiol 2014;29:363-9.

4. Nguyen S, Baggerly L, French C, Heaney RP, Gorham ED, Garland CF. 25-Hydroxyvitamin D in the range of 20 to 100 ng/mL and incidence of kidney stones. American journal of public health 104 (9) (pp 1783-1787), 2014;Sep.

5. Cho YS, Joo KJ, Park HJ, Kwon CH. Relationship between nephrolithiasis and components of metabolic syndrome in Korean adults. European Urology, Supplements Conference: 25th Anniversary Congress of the European Association of Urology, EAU Barcelona Spain Conference Publication: (var pagings) 9 (2) (pp 168), 2010;April.

6. Lieske JC, Mehta RA, Milliner DS, Rule AD, Bergstralh EJ, Sarr MG. Kidney stones are common after bariatric surgery. Kidney International 87 (4) (pp 839-845), 2015;08.

7. Hamano S, Nakatsu H, Suzuki N, Tomioka S, Tanaka M, Murakami S. Kidney stone disease and risk factors for coronary heart disease. Int J Urol 2005;12:859-63.

8. Lieske JC, de l, V, Gettman MT et al. Diabetes mellitus and the risk of urinary tract stones: a population-based case-control study. Am J Kidney Dis 2006;48:897-904.

9. Daudon M, Traxer O, Conort P, Lacour B, Jungers P. Type 2 diabetes increases the risk for uric acid stones. J Am Soc Nephrol 2006;17:2026-33.

10. Milicevic S, Bijelic R, Krivokuca V, Bojic M, Popovic-Pejicic S, Bojanic N. Correlation of the body mass index and calcium nephrolithiasis in adult population. Med Arch 2013;67:423-7.

11. Wang J, Luo GT, Niu WJ et al. [Risk factors for the kidney stones: a hospital-based case-control study in a distric hospital in Beijing]. Beijing Da Xue Xue Bao 2013;45:971-4.

12. Nerli R, Jali M, Guntaka AK, Patne P, Patil S, Hiremath MB. Type 2 diabetes mellitus and renal stones. Adv Biomed Res 2015;4:180.

13. Akarken I, Tarhan H, Ekin RG et al. Visceral obesity: A new risk factor for stone disease. Can Urol Assoc J 2015;9:E795-E799.

14. Zhao A, Dai M, Chen YJ, Chang HE, Liu AP, Wang PY. Risk factors associated with nephrolithiasis: a case-control study in China. Asia Pac J Public Health 2015;27:NP414-NP424.

15. Wu CC, Hung SH, Lin HC, Lee CZ, Lee HC, Chung SD. Sialolithiasis is associated with nephrolithiasis: a case-control study. Acta Otolaryngol 2016;136:497-500.

16. Xiao X, Dong Z, Ye X et al. Association between OPN genetic variations and nephrolithiasis risk. Biomed Rep 2016;5:321-6.

17. Peng J, Zhou HB, Cheng JQ, Dong SF, Shi LY, Zhang D. [Study on the epidemiology and risk factors of renal calculi in special economic zone of Shenzhen city]. Zhonghua Liu Xing Bing Xue Za Zhi 2003;24:1112-4.

18. Verde I, Rusu E, Suliman E, Costache A, Armean P. Diabetes in the hospitalized patients with urological diseases. J Med Life 2015;8:496-501.

19. Hall WD, Pettinger M, Oberman A et al. Risk factors for kidney stones in older women in the southern United States. Am J Med Sci 2001;322:12-8.

20. Arzoz M, Ibarz L, Roca J et al. Relationship between metabolic syndrome and kidney stone: Differences between first time and recurrent stone formers. European Urology, Supplements Conference: 26th Annual Congress of the European Association of Urology, EAU Vienna Austria Conference Publication: (var pagings) 10 (2) (pp 228), 2011;March.

21. Dai M, Zhao A, Liu A, You L, Wang P. Dietary Factors and Risk of Kidney Stone: A Case-Control Study in Southern China. Journal of Renal Nutrition 23 (2) (pp e21-e28), 2013;March.

22. Sancak EB, Resorlu M, Akbas A, Gulpinar MT, Arslan M, Resorlu B. Do hypertension, diabetes mellitus and obesity increase the risk of severity of nephrolithiasis? Pakistan Journal of Medical Sciences 31 (3) (pp 566-571), 2015;2015.

23. Ticinesi A, Nouvenne A, Ferraro PM et al. Idiopathic Calcium Nephrolithiasis and Hypovitaminosis D: A Case-control Study. Urology 87 (pp 40-45), 2016;01.

24. Mosli HA, Mosli HH. Increased body mass index is associated with larger renal calculi. Urology 2012;80:974-7.

25. Inci M, Demirtas A, Sarli B, Akinsal E, Baydilli N. Association between body mass index, lipid profiles, and types of urinary stones. Ren Fail 2012;34:1140-3.

26. Kadlec AO, Greco K, Fridirici ZC, Hart ST, Vellos T, Turk TM. Metabolic syndrome and urinary stone composition: what factors matter most? Urology 2012;80:805-10.

27. Torricelli FC, De S, Gebreselassie S, Li I, Sarkissian C, Monga M. Type-2 diabetes and kidney stones: impact of diabetes medications and glycemic control. Urology 2014;84:544-8.

28. Torricelli FC, De SK, Gebreselassie S, Li I, Sarkissian C, Monga M. Dyslipidemia and kidney stone risk. J Urol 2014;191:667-72.

29. Trinchieri A, Croppi E, Montanari E. Obesity and urolithiasis: evidence of regional influences. Urolithiasis 2017;45:271-8.

30. Yi JJ, Yi LJ, Xiang GZ, Qing WW, Yong LJ, Jiang LJ. Re: The association of increasing body mass index and kidney stone disease. M. J. Semins, A. D. Shore, M. A. Makary, T. Magnuson, R. Johns and B. R. Matlaga. J Urol 2010; 183: 571-575. J Urol 2011;185:1984.

31. Iguchi T, Nakatani T. Editorial comment to percutaneous nephrolithotomy increases the risk of diabetes: a 5-year follow-up study. Int J Urol 2014;21:669.

32. Lieske JC. New insights regarding the interrelationship of obesity, diet, physical activity, and kidney stones. J Am Soc Nephrol 2014;25:211-2.

33. Soucie JM, Coates RJ, McClellan W, Austin H, Thun M. Relation between geographic variability in kidney stones prevalence and risk factors for stones. Am J Epidemiol 1996;143:487-95.

34. Ramey SL, Franke WD, Shelley MC. Relationship among risk factors for nephrolithiasis, cardiovascular disease, and ethnicity: focus on a law enforcement cohort. AAOHN J 2004;52:116-21.

35. West B, Luke A, Durazo-Arvizu RA, Cao G, Shoham D, Kramer H. Metabolic syndrome and self-reported history of kidney stones: the National Health and Nutrition Examination Survey (NHANES III) 1988-1994. Am J Kidney Dis 2008;51:741-7.

36. West B, Luke A, Durazo-Arvizu RA, Cao G, Shoham D, Kramer H. Metabolic Syndrome and Self-Reported History of Kidney Stones: The National Health and Nutrition Examination Survey (NHANES III) 1988-1994. American Journal of Kidney Diseases 51 (5) (pp 741-747), 2008;May.

37. Rendina D, Mossetti G, De FG et al. Association between metabolic syndrome and nephrolithiasis in an inpatient population in southern Italy: role of gender, hypertension and abdominal obesity. Nephrol Dial Transplant 2009;24:900-6.

38. Semins MJ, Shore AD, Makary MA, Magnuson T, Johns R, Matlaga BR. The association of increasing body mass index and kidney stone disease. J Urol 2010;183:571-5.

39. Kohjimoto Y, Sasaki Y, Iguchi M, Inagaki T, Hara I. Metabolic syndrome increases the risk for kidney stones: RESULTS from a Nationwide Survey on urolithiasis in Japan. Journal of Urology Conference: 2010;April.

40. Nowfar S, Palazzi-Churas K, Chang DC, Sur RL. The relationship of obesity and gender prevalence changes in United States inpatient nephrolithiasis. Urology 2011;78:1029-33.

41. Jeong IG, Kang T, Bang JK et al. Association between metabolic syndrome and the presence of kidney stones in a screened population. Am J Kidney Dis 2011;58:383-8.

42. Ando R, Suzuki S, Nagaya T et al. Impact of insulin resistance, insulin and adiponectin on kidney stones in the Japanese population. Int J Urol 2011;18:131-8.

43. Kabeya Y, Kato K, Tomita M et al. Associations of insulin resistance and glycemic control with the risk of kidney stones. Intern Med 2012;51:699-705.

44. Kim YJ, Kim CH, Sung EJ, Kim SR, Shin HC, Jung WJ. Association of nephrolithiasis with metabolic syndrome and its components. Metabolism 2013;62:808-13.

45. Ando R, Nagaya T, Suzuki S et al. Kidney stone formation is positively associated with conventional risk factors for coronary heart disease in Japanese men. J Urol 2013;189:1340-6.

46. Weinberg AE, Patel CJ, Chertow GM, Leppert JT. Diabetic severity and risk of kidney stone disease. Eur Urol 2014;65:242-7.

47. Patil SM, Nerli RB, Jali MV, Guntaka AK, Patne PB, Hiremath MB. Type 2 diabetes mellitus and renal stones. Indian Journal of Urology Conference: 47th Annual Conference of Urological Society of India, USICON 2014;January.

48. Daels FP, Gaizauskas A, Rioja J et al. Age-related prevalence of diabetes mellitus, cardiovascular disease and anticoagulation therapy use in a urolithiasis population and their effect on outcomes: the Clinical Research Office of the Endourological Society Ureteroscopy Global Study. World J Urol 2015;33:859-64.

49. Cano-Castineira R, Carrasco-Valiente J, Perula-de-Torres LA et al. Prevalence of renal stones in Andalusian population: results of PreLiRenA study. Actas Urol Esp 2015;39:26-31.

50. Mello MF, Marchini GS, Camara C et al. A large 15 - year database analysis on the influence of age, gender, race, obesity and income on hospitalization rates due to stone disease. Int Braz J Urol 2016;42:1150-9.

51. Moudi E, Hosseini SR, Bijani A. Nephrolithiasis in elderly population; effect of demographic characteristics. J Nephropathol 2017;6:63-8.

52. Zeng G, Mai Z, Xia S et al. Prevalence of kidney stones in China: an ultrasonography based cross-sectional study. BJU Int 2017;120:109-16.

53. Liu Y-T, Yang P-Y, Yang Y-W, Sun H-Y, Lin I-C. The association of nephrolithiasis with metabolic syndrome and its components: A cross-sectional analysis. Therapeutics and Clinical Risk Management 13 (pp 41-48), 2017;2017.

54. Arias VR, Perula de Torres LA, Jimenez GC et al. Medicina Clinica (no pagination), 2017;December.

55. Sancak EB, Resorlu M, Akbas A, Gulpinar MT, Arslan M, Resorlu B. Do Hypertension, diabetes mellitus and obesity increase the risk of severity of nephrolithiasis? Pak J Med Sci 2015;31:566-71.

56. Hassan EM, Malekpour Z, Pourrezaei F, Ghaem H. Prevalence of obesity and abdominal obesity in patients with kidney stone disease. Journal of Gastroenterology and Hepatology Conference: Asian Pacific Digestive Week 2012;December.

57. Scales C, Smith A, Hanley J, Saigal C. Obesity, socioeconomic status, and the risk of kidney stones in the united states. Journal of Urology Conference: 2012;April.

58. Kohjimoto Y, Sasaki Y, Iguchi M, Matsumura N, Inagaki T, Hara I. Association of metabolic syndrome traits and severity of kidney stones: Results from a nationwide survey on urolithiasis in Japan. American Journal of Kidney Diseases 61 (6) (pp 923-929), 2013;June.

59. Gordiano EA, Tondin LM, Miranda RC, Baptista DR, Carvalho M. Evaluation of food intake and excretion of metabolites in nephrolithiasis. Jornal brasileiro de nefrologia : 'orgao oficial de Sociedades Brasileira e Latino-Americana de Nefrologia 36 (4) (pp 437-445), 2014;01.

60. Pigna F, Sakhaee K, ms-Huet B, Maalouf NM. Body fat content and distribution and urinary risk factors for nephrolithiasis. Clinical Journal of the American Society of Nephrology 9 (1) (pp 159-165), 2014;07.

61. Weinberg A, Patel C, Chertow G, Leppert J. Diabetic severity and risk of kidney stone disease. Journal of Urology Conference: 2013;April.

62. Curhan GC, Willett WC, Rimm EB, Speizer FE, Stampfer MJ. Body size and risk of kidney stones. J Am Soc Nephrol 1998;9:1645-52.

63. Semins MJ, Shore AD, Steele K, Makary MA, Matlaga BR. The effect of increasing body mass index on kidney stone disease. Journal of Urology Conference: 2009;var.

64. Sorensen MD, Kahn AJ, Reiner AP et al. Impact of nutritional factors on incident kidney stone formation: a report from the WHI OS. J Urol 2012;187:1645-9.

65. Ferraro PM, Taylor EN, Gambaro G, Curhan GC. Dietary and Lifestyle Risk Factors Associated with Incident Kidney Stones in Men and Women. J Urol 2017.

66. Cappuccio FP, Siani A, Barba G et al. A prospective study of hypertension and the incidence of kidney stones in men. Journal of Hypertension 17 (7) (pp 1017-1022), 1999;1999.

67. Sarica K, Eryildirim B, Yencilek F, Kuyumcuoglu U. Role of Overweight Status on Stone-forming Risk Factors in Children: A Prospective Study. Urology 73 (5) (pp 1003-1007), 2009;May.

68. Rezaee ME, Ward CE, Pollock M, Shetty SD. Association between multiple chronic conditions and urolithiasis. Int Urol Nephrol 2017.

69. Indridason OS, Birgisson S, Edvardsson VO, Sigvaldason H, Sigfusson N, Palsson R. Epidemiology of kidney stones in Iceland: a population-based study. Scand J Urol Nephrol 2006;40:215-20.

70. Sarica K, Altay B, Erturhan S. Effect of Being Overweight on Stone-Forming Risk Factors. Urology 71 (5) (pp 771-774), 2008;May.

71. Weikert C, Weikert S, Schulze MB et al. Presence of gallstones or kidney stones and risk of type 2 diabetes. Am J Epidemiol 2010;171:447-54.

72. Tiwari R, Campfield T, Wittcopp C et al. Metabolic syndrome in obese adolescents is associated with risk for nephrolithiasis. J Pediatr 2012;160:615-20.

73. Shavit L, Ferraro PM, Johri N et al. Effect of being overweight on urinary metabolic risk factors for kidney stone formation. Nephrology Dialysis Transplantation 30 (4) (pp 607-613), 2015;01.

74. Ando R, Suzuki S, Nagaya T et al. Impact of insulin resistance, serum insulin and adiponectin levels on kidney stones in Japanese population. Journal of Urology Conference: 2010;April.

75. Assimos DG. Re: Ascorbic acid supplements and kidney stone incidence among men: A prospective study. Journal of Urology 190;October.

76. Taylor EN, Curhan GC. Dietary calcium from dairy and nondairy sources, and risk of symptomatic kidney stones. Journal of Urology 190;October.

77. Shoag J, Eisner BH. Relationship between C-reactive protein and kidney stone prevalence. Journal of Urology 191;2014.

78. Baber RJ. Postmenopausal hormone use and the risk of nephrolithiasis. Climacteric 14 (1) (pp 191;February.

79. Ferraro PM, Taylor EN, Gambaro G, Curhan GC. Vitamin D Intake and the Risk of Incident Kidney Stones. Journal of Urology 197;01.

80. Taylor EN, Curhan GC. Fructose consumption and the risk of kidney stones. Kidney International 73 (2) (pp 207;10.

81. La VC, Decarli A, Pagano R. Vegetable consumption and risk of chronic disease. Epidemiology 9 (2) (pp 208;March.

82. Holoch PA, Tracy CR. Antioxidants and self-reported history of kidney stones: The national health and nutrition examination survey. Journal of Endourology 25 (12) (pp 1903;01.

83. Van MT, Tielemans C, Wissing KM, Vonckx L. Risk factors for kidney stones and the impact of preventive measures. A retrospective cohort study of patients attending an outpatient kidney stone clinic. Acta Clinica Belgica: International Journal of Clinical and Laboratory Medicine Conference: 1920;2015.

84. Taylor EN, Fung TT, Curhan GC. DASH-style diet associates with reduced risk for kidney stones. Journal of the American Society of Nephrology 1920;October.

85. Power C, Barker DJP, Nelson M, Winter PD. Diet and renal stones: A case-control study. British Journal of Urology 56 (5) (pp 456-459), 1984;1984.

86. Kramer HM, Curhan G. The association between gout and nephrolithiasis: The National Health and Nutrition Examination Survey III, 1988-1994. American Journal of Kidney Diseases 40 (1) (pp 37-42), 2002;2002.

87. Kramer HJ, Choi HK, Atkinson K, Stampfer M, Curhan GC. The association between gout and nephrolithiasis in men: The Health Professionals' Follow-Up Study. Kidney International 64 (3) (pp 1022-1026), 2003;01.

88. Taylor EN, Stampfer MJ, Curhan GC. Dietary factors and the risk of incident kidney stones in men: New insights after 14 years of follow-up. Journal of the American Society of Nephrology 15 (12) (pp 3225-3232), 2004;December.

89. Taylor EN, Curhan GC. Oxalate intake and the risk for nephrolithiasis. Journal of the American Society of Nephrology 18 (7) (pp 2198-2204), 2007;July.

90. Eisner BH, Eisenberg ML, Stoller ML. Impact of Urine Sodium on Urine Risk Factors for Calcium Oxalate Nephrolithiasis. Journal of Urology 182 (5) (pp 2330-2333), 2009;November.

91. Semins MJ, Matlaga BR, Shore AD et al. The Effect of Gastric Banding on Kidney Stone Disease. Urology 74 (4) (pp 746-749), 2009;October.

92. Matlaga BR, Shore AD, Magnuson T, Clark JM, Johns R, Makary MA. Effect of Gastric Bypass Surgery on Kidney Stone Disease. Journal of Urology 181 (6) (pp 2573-2577), 2009;June.

93. Robusto F, Colucci G, Iacovazzo P et al. Food quality and co-morbidity in the kidney stones: Observational case-control study. Archivio Italiano di Urologia e Andrologia Conference: 6th Eurolithiasis (eULIS) Society Symposium 14th European Symposium on Urolithiasis Como Italy Conference Publication: (var pagings) 81 (3) (pp 128), 2009;September.

94. Rentschler A, Oehlschlager S, Wirth MP. Nutritional, environmental and family history determinants of recurrent nephrolithiasis versus primary nephrolithiasis. European Urology, Supplements Conference: 25th Anniversary Congress of the European Association of Urology, EAU Barcelona Spain Conference Publication: (var pagings) 9 (2) (pp 168), 2010;April.

95. Assimos D, Knight J, Holmes R. Impact of dietary fructose on kidney stone risk. Journal of Urology Conference: 2010;April.

96. Holmes RP, Knight J, Mufarij P, Assimos DG. Impact of dietary fructose on stone risk parameters. Journal of Endourology Conference: 28th World Congress of Endourology and SWL Chicago, IL United States Conference Publication: (var pagings) 24 (pp A6), 2010;September.

97. Sorensen MD, Eisner BH, Stone KL et al. Fractional calcium absorption is associated with nephrolithiasis in older women in the Study of Osteoporotic Fractures (SOF). Journal of the American College of Surgeons Conference: 97th Annual Clinical Congress of the American College of Surgeons San Francisco, CA United States Conference Publication: (var pagings) 213 (3 SUPPL 1) (pp S145), 2011;September.

98. Tang J, McFann KK, Chonchol MB. Association between serum 25-hydroxyvitamin D and nephrolithiasis: The National Health and Nutrition Examination Survey III, 1988-94. Nephrology Dialysis Transplantation 27 (12) (pp 4385-4389), 2012;December.

99. Scheffler P, Dunn M, Christou NV, Andersen RE, Andonian S. Increasing doses of vitamin d supplementation are associated with decreased risk of nephrolithiasis in post gastric bypass surgery. Journal of Urology Conference: 2012;April.

100. Ferraro PM, Taylor EN, Gambaro G, Curhan GC. Soda and other beverages and the risk of kidney stones. Clinical Journal of the American Society of Nephrology 8 (8) (pp 1389-1395), 2013;July.

101. Tan M, Xia S, Zhang Q, Zhu J, Bao E. The -160C>a polymorphism in E-cadherin is associated with the risk of nephrolithiasis. PloS one 8 (9) (pp e73109), 2013;2013.

102. Masterson JH, Woo JR, Chang DC, L'Esperance JO, Stoller ML, Sur RL. Hyperlipidemia is associated with an increased risk of nephrolithiasis LCDR. Journal of Endourology Conference: 31st World Congress of Endourology and SWL, WCE 2013;October.

103. Sorensen M, Hsi R, Chi T et al. Dietary intake of fiber, vegetables, and fruit decrease the risk of incident kidney stones in women: A women's health initiative (WHI) report. Journal of Urology Conference: 2013;April.

104. Masterson LCDR, Woo JR, Chang DC, L'Esperance CDRJ, Stoller ML, Sur RL. Do statin medications reduce the incidence of nephrolithiasis in patients with hyperlipidemia? Journal of Urology Conference: 2013;April.

105. Li C-H, Sung F-C, Wang Y-C, Lin D, Kao C-H. Gallstones increase the risk of developing renal stones: A nationwide population-based retrospective cohort study. QJM 107 (6) (pp 451-457), 2014;hcu017.

106. Ferraro PM, Taylor EN, Gambaro G, Curhan GC. Caffeine intake and the risk of kidney stones. American Journal of Clinical Nutrition 100 (6) (pp 1596-1603), 2014;01.

107. Oddsson A, Sulem P, Helgason H et al. Common and rare variants associated with kidney stones and biochemical traits. Nature Communications 6 (no pagination), 2015;7975.

108. Loftus CJ, Byrne MD, Sivalingam S. Increased rate of kidney stone formation with calcium supplementation after bariatric surgery. Endocrine Reviews Conference: 97th Annual Meeting and Expo of the Endocrine Society, ENDO 2015;2015.

109. Taylor EN, Hoofnagle AN, Curhan GC. Calcium and phosphorus regulatory hormones and risk of incident symptomatic kidney stones. Clinical Journal of the American Society of Nephrology 10 (4) (pp 667-675), 2015;2015.

110. Park S, Fahey N, Lapin B, Pruitt J, Wang C. Vitamin D deficiency treated with weekly 50,000 IU vitamin D supplementation increases renal stone risk. Journal of Urology Conference: 2015;April.

111. Ferraro PM, Curhan GC, Gambaro G, Taylor EN. Total, dietary, and supplemental Vitamin C intake and risk of incident kidney stones. American Journal of Kidney Diseases 67 (3) (pp 400-407), 2016;01.

112. Hara A, Yang W-Y, Petit T et al. Incidence of nephrolithiasis in relation to environmental exposure to lead and cadmium in a population study. Environmental Research 145 (pp 1-8), 2016;February.

113. Ma G, Yuan Q, Wang Q et al. Association between MIF-AS rs755622 and nephrolithiasis risk in a Chinese population. Medical Science Monitor 22 (pp 563-568), 2016;20.

114. Cohen A, Adamsky M, Nottingham C, Pruitt J, Lapin B, Park S. Statin intake reduces kidney stone formation. Journal of Urology Conference: 2016;April.

115. Tsai S-H, Stoller M, Sherer B, Chao Z-H, Tong T-H. Sleep apnea: An independent risk factor for nephrolithiasis. Journal of Urology Conference: 112th Annual Meeting of the American Urological Association, AUA 2017;April.

116. Tasian G, Jemelieta T, Goldfarb D et al. Association between oral antibiotics and incident kidney stones. Journal of Urology Conference: 112th Annual Meeting of the American Urological Association, AUA 2017;April.

117. Ferraro PM, Mandel EI, Curhan GC, Gambaro G, Taylor EN. Dietary protein and potassium, diet-dependent net acid load, and risk of incident kidney stones. Clinical Journal of the American Society of Nephrology 11 (10) (pp 1834-1844), 2016;07.

118. Saldana TM, Basso O, Darden R, Sandler DP. Carbonated beverages and chronic kidney disease. Epidemiology 18 (4) (pp 501-506), 2007;July.

119. Saucier NA, Sinha MK, Liang KV et al. Risk Factors for CKD in Persons With Kidney Stones: A Case-Control Study in Olmsted County, Minnesota. American Journal of Kidney Diseases 55 (1) (pp 61-68), 2010;January.

120. Purnomo AD, Rochadi S, Indrawarman. The influence of body mass index (BMI) for stone free rate, complication, and length of stay patient post percutaneous nephrolithotomy (PCNL). BJU International Conference: 37th Annual Scientific Meeting of the Indonesian Urological Association Lombok Indonesia Conference Publication: (var pagings) 114 (pp 12), 2014;October.

121. Zhou T, Watts K, Agalliu I, DiVito J, Hoenig DM. Effects of visceral fat area and other metabolic parameters on stone composition in patients undergoing percutaneous nephrolithotomy. J Urol 2013;190:1416-20.

122. Ayoob R, Wang W, Schwaderer A. Body fat composition and occurrence of kidney stones in hypercalciuric children. Pediatr Nephrol 2011;26:2173-8.

123. Vescini F, Buffa A, Caudarella R. The influence of calcium on renal stone disease. [Italian]. Progress in Nutrition 9 (1) (pp 1920;2007.

124. Saxena AK. Emerging global epidemic of obesity: the renal perspective. Ann Saudi Med 2006;26:288-95.

125. Rutkowski P, Klassen A, Sebekova K, Bahner U, Heidland A. Renal disease in obesity: the need for greater attention. J Ren Nutr 2006;16:216-23.

126. Daudon M, Jungers P. Diabetes and nephrolithiasis. Curr Diab Rep 2007;7:443-8.

127. Ferrari P, Piazza R, Ghidini N, Bisi M, Galizia G, Ferrari G. Lithiasis and risk factors. Urol Int 2007;79 Suppl 1:8-15.

128. Eknoyan G. Obesity, diabetes, and chronic kidney disease. Current Diabetes Reports 7 (6) (pp 449-453), 2007;December.

129. Mossetti G, Rendina D, De FG et al. Metabolic syndrome and nephrolithiasis: can we hypotize a common background? Clin Cases Miner Bone Metab 2008;5:114-7.

130. Trinchieri A. Epidemiology of urolithiasis: an update. Clin Cases Miner Bone Metab 2008;5:101-6.

131. Obligado SH, Goldfarb DS. The association of nephrolithiasis with hypertension and obesity: a review. Am J Hypertens 2008;21:257-64.

132. Frassetto L, Kohlstadt I. Treatment and prevention of kidney stones: an update. Am Fam Physician 2011;84:1234-42.

133. Ahmed MH, Ahmed HT, Khalil AA. Renal stone disease and obesity: what is important for urologists and nephrologists? Ren Fail 2012;34:1348-54.

134. Morandi A, Maffeis C. Urogenital complications of obesity. Best Pract Res Clin Endocrinol Metab 2013;27:209-18.

135. Alicic RZ, Patakoti R, Tuttle KR. Direct and indirect effects of obesity on the kidney. Adv Chronic Kidney Dis 2013;20:121-7.

136. Ramaswamy K, Shah O. Metabolic syndrome and nephrolithiasis. Transl Androl Urol 2014;3:285-95.

137. Naguib MT. Kidney disease in the obese patient. South Med J 2014;107:481-5.

138. Domingos F, Serra A. Metabolic syndrome: a multifaceted risk factor for kidney stones. Scand J Urol 2014;48:414-9.

139. Roudakova K, Monga M. The evolving epidemiology of stone disease. Indian J Urol 2014;30:44-8.

140. Sakhaee K. Epidemiology and clinical pathophysiology of uric acid kidney stones. J Nephrol 2014;27:241-5.

141. DiBianco JM, Jarrett TW, Mufarrij P. Metabolic Syndrome and Nephrolithiasis Risk: Should the Medical Management of Nephrolithiasis Include the Treatment of Metabolic Syndrome? Rev Urol 2015;17:117-28.

142. Manoj Kumar KR. Weight gain and the risk of kidney stones. Journal of Pharmaceutical Sciences and Research 7 (9) (pp 776-778), 2015;01.

143. Prochaska ML, Taylor EN, Curhan GC. Insights Into Nephrolithiasis From the Nurses' Health Studies. Am J Public Health 2016;106:1638-43.

144. Ozgor F, Ucpinar B, Binbay M. Effect of Obesity on Prone Percutaneous Nephrolithotomy Outcomes: A Systemic Review. Urol J 2016;13:2471-8.

145. Wong Y, Cook P, Roderick P, Somani BK. Metabolic Syndrome and Kidney Stone Disease: A Systematic Review of Literature. J Endourol 2016;30:246-53.

146. Kovesdy CP, Furth L, Zoccali C. Obesity and kidney disease: hidden consequences of the epidemic. Clin Kidney J 2017;10:1-8.

147. Kovesdy CP, Furth S, Zoccali C. Obesity and kidney disease: hidden consequences of the epidemic. Rev Med Chil 2017;145:281-91.

148. Kovesdy CP, Furth SL, Zoccali C. Obesity and Kidney Disease: Hidden Consequences of the Epidemic. Can J Kidney Health Dis 2017;4:2054358117698669.

149. Kovesdy CP, Furth SL, Zoccali C. Obesity and kidney disease: hidden consequences of the epidemic. Braz J Med Biol Res 2017;50:e6075.

150. Kovesdy CP, Furth S, Zoccali C, World Kidney Day Steering Committee. Obesity and kidney disease: Hidden consequences of the epidemic. Physiol Int 2017;104:1-14.

151. Kovesdy CP, Furth SL, Zoccali C. Obesity and kidney disease: hidden consequences of the epidemic. J Bras Nefrol 2017;39:1-10.

152. Kovesdy CP, Furth S, Zoccali C. Obesity and kidney disease: Hidden consequences of the epidemic. Saudi J Kidney Dis Transpl 2017;28:241-52.

153. Kovesdy CP, Furth S, Zoccali C. Obesity and kidney disease: Hidden consequences of the epidemic. Arch Esp Urol 2017;70:311-21.

154. Kovesdy CP, Furth S, Zoccali C, World Kidney Day Steering Committee /. Obesity and Kidney Disease: Hidden Consequences of the Epidemic. Iran J Kidney Dis 2017;11:99-108.

155. Kovesdy CP, Furth SL, Zoccali C. Obesity and Kidney Disease: Hidden Consequences of the Epidemic. Blood Purif 2017;43:346-54.

156. Kovesdy CP, Furth SL, Zoccali C. Obesity and Kidney Disease: Hidden Consequences of the Epidemic. J Ren Nutr 2017;27:75-7.

157. Kovesdy CP, Furth SL, Zoccali C. Obesity and kidney disease: hidden consequences of the epidemic. J Nephrol 2017;30:1-10.

158. Kovesdy CP, Furth SL, Zoccali C. Obesity and kidney disease: Hidden consequences of the epidemic. J Ren Care 2017;43:3-10.

159. Kovesdy CP, Furth SL, Zoccali C. Obesity and Kidney Disease: Hidden Consequences of the Epidemic. Am J Nephrol 2017;45:283-91.

160. Kovesdy CP, Furth SL, Zoccali C. Obesity and Kidney Disease: Hidden Consequences of the Epidemic. Nephron 2017;135:243-51.

161. Kovesdy CP, Furth S, Zoccali C. Obesity and kidney disease: Hidden consequences of the epidemic. Nephrol Ther 2017;13:131-7.

162. Kovesdy CP, Furth SL, Zoccali C. Obesity and kidney disease: hidden consequences of the epidemic. Kidney Int 2017;91:260-2.

163. Kovesdy CP, Furth S, Zoccali C et al. Obesity and kidney disease: Hidden consequences of the epidemic. [Spanish]. Revista de Nefrologia, Dialisis y Trasplante 37 (1) (pp 1-12), 2017;2017.

164. Kovesdy CP, Furth SL, Zoccali C. Obesity and Kidney Disease: Hidden Consequences of the Epidemic. Kidney Dis (Basel) 2017;3:33-41.

165. Hyams E, Dulabon L, Shah O. Obesity and the treatment of nephrolithiasis: A systematic review of the literature and meta-analysis. Journal of Endourology Conference: 27th WCE 2009;October.

166. Rendina D, De FG, D'Elia L, Strazzullo P. Metabolic syndrome and nephrolithiasis: A systematic review and meta-analysis of the scientific evidence. Journal of Nephrology 27 (4) (pp 371-376), 2014;August.

Supplementary Table 2. Prospective studies of adiposity and kidney stones

First author, publication year, country	Study name or description	Study period	Number of participants, number of cases	Measure-ment of anthro-pometry	Exposure	Comparison	Relative risk (95% confidence interval)	Adjustment for confounders
Taylor EN et al, 2005, USA	Health Professionals Follow-up Study	1986-2002, 16 years follow-up	45988 men, age 40-75 years:<br>1609 cases<br>1483 cases (weight change)<br>1214 cases (waist circumference)	Self-reported (validated)	Weight<br><br><br><br><br>Weight change between age 21 years and baseline<br><br><br><br><br>BMI<br><br><br><br><br><br>Waist circumference	64.5 kg<br>72.7<br>80.9<br>90.9<br>106.8<br>-5.9 kg<br>0<br>+5.0 kg<br>+11.4 kg<br>+15.9 kg<br><21.0<br>21.0-22.9<br>23.0-24.9<br>25.0-27.4<br>27.5-29.9<br>≥30.0<br>86.4 cm<br>90.2<br>96.5<br>104.1<br>114.3	1.00<br>1.15 (0.93-1.43)<br>1.34 (1.09-1.66)<br>1.33 (1.07-1.65)<br>1.44 (1.11-1.86)<br>1.05 (0.81-1.36)<br>1.00<br>1.24 (1.03-1.49)<br>1.27 (1.04-1.53)<br>1.39 (1.14-1.70)<br>0.73 (0.52-1.03)<br>1.00<br>0.94 (0.79-1.12)<br>1.20 (1.02-1.41)<br>1.24 (1.03-1.50)<br>1.33 (1.08-1.63)<br>1.00<br>1.23 (1.00-1.51)<br>1.37 (1.11-1.69)<br>1.42 (1.13-1.78)<br>1.48 (1.13-1.93)	Age, use of thiazide diuretics, alcohol, calcium supplement use, fluids, animal protein, calcium, magnesium, potassium, sodium, vitamin C<br>Weight change: also adjusted for weight in early adulthood
Taylor EN et al, 2005, USA	Nurses' Health Study 1	1980-2000, 20 years follow-up	93758 women, age 34-59 years:<br>1687 cases<br>1580 cases (weight change)<br>1108 cases (waist circumference)	Self-reported (validated)	Weight<br><br><br><br><br>Weight change between age 18 years and baseline<br><br><br><br><br>BMI	60.0 kg<br>71.8<br>80.5<br>90.9<br>109.1<br>-6.4 kg<br>0<br>+5.5<br>+11.8<br>+22.7<br><21.0	1.00<br>1.29 (1.14-1.46)<br>1.37 (1.18-1.59)<br>1.61 (1.37-1.89)<br>1.89 (1.52-2.36)<br>0.93 (0.71-1.23)<br>1.00<br>1.00 (0.82-1.22)<br>1.23 (1.01-1.50)<br>1.70 (1.46-2.05)<br>1.15 (0.95-1.38)	Age, use of thiazide diuretics, alcohol, calcium supplement use, fluids, animal protein, calcium, magnesium, potassium, sodium, vitamin C<br>Weight change: also adjusted for weight in early adulthood

						21.0-22.9<br>23.0-24.9<br>25.0-27.4<br>27.5-29.9<br>≥30.0	1.00<br>1.26 (1.07-1.50)<br>1.34 (1.13-1.59)<br>1.75 (1.45-2.10)<br>1.90 (1.61-2.25)	
					Waist circumference	72.4 cm<br>81.3<br>88.9<br>96.5<br>109.2	1.00<br>1.24 (1.05-1.47)<br>1.44 (1.21-1.72)<br>1.49 (1.23-1.80)<br>1.71 (1.40-2.10)	
Taylor EN et al, 2005, USA	Nurses' Health Study 2	1991-2001, 10 years follow-up	101877 women, age 27-44 years: 1531 cases<br>1512 cases (weight change)<br>751 cases (waist circumference)	Self-reported (validated)	Weight	59.1 kg<br>70.9<br>80.5<br>90.9<br>111.4	1.00<br>1.26 (1.10-1.45)<br>1.49 (1.27-1.75)<br>1.73 (1.47-2.04)<br>1.92 (1.59-2.31)	Age, use of thiazide diuretics, alcohol, calcium supplement use, fluids, animal protein, calcium, magnesium, potassium, sodium, vitamin C
					Weight change between age 18 years and baseline	-5.5 kg<br>0<br>+4.5<br>+11.4<br>+22.7	1.13 (0.86-1.50)<br>1.00<br>1.03 (0.84-1.26)<br>1.27 (1.03-1.55)<br>1.82 (1.50-2.21)	Weight change: also adjusted for weight in early adulthood
					BMI	<21.0<br>21.0-22.9<br>23.0-24.9<br>25.0-27.4<br>27.5-29.9<br>≥30.0	1.13 (0.94-1.36)<br>1.00<br>1.24 (1.04-1.50)<br>1.47 (1.22-1.77)<br>1.68 (1.36-2.07)<br>2.09 (1.77-2.48)	
					Waist circumference	71.1 cm<br>81.3<br>88.9<br>96.5<br>111.8	1.00<br>0.98 (0.80-1.21)<br>1.21 (0.96-1.54)<br>1.71 (1.34-2.17)<br>1.94 (1.49-2.52)	
Akoudad S, 2010, USA	Atherosclerosis Risk in Communities Study	1993-1995 - 2005, 10.8 years follow-up	12161 men and women, age 45-64 years: 971 cases	Measured	Waist circumference	Per 14.4 cm	1.11 (0.88-1.40)	Age, sex, race, region, diabetes, triglycerides, hypertension, uric acid, gallstones
Oda E et al, 2014, Japan	Medical Check-up Center,	2008-2009 - 2013, 3.2	1726 men and 992 women,	Measured	BMI, men<br>BMI, women	Per 1 unit<br>Per 1 unit	1.042 (0.987-1.101)<br>0.949 (0.866-1.040)	Age, antihypertensive drug use, antidiabetic or antihyperlipidemic

	Nagaoka	years follow-up	mean age 51.9 years: 238 cases		Waist circumference, men<br>Waist circumference, women	Per 1 cm<br><br>Per 1 cm	1.015 (0.996-1.035)<br><br>0.988 (0.958-1.019)	drug use, smoking, alcohol, physical activity, history of coronary heart disease and stroke, hs-CRP, SBP, fasting glucose, TG, HDL cholesterol, LDL cholesterol, uric acid, sodium, potassium, chloride, calcium
Sorensen MD et al, 2014, USA	Women's Health Initiative	1993-1998 - NA, 8 years follow-up	84225 women, age 50-79 years: 2392 cases	Measured	BMI<br><br><br><br><br>BMI	<18.5<br>18.5-24.9<br>25.0-29.9<br>30.0-34.9<br>≥35.0<br><18.5<br>18.5-24.9<br>25.0-29.9<br>30.0-34.9<br>≥35.0	0.79 (0.47-1.32)<br>1.00<br>1.30 (1.17-1.44)<br>1.62 (1.43-1.83)<br>1.81 (1.57-2.10)<br>0.79 (0.47-1.34)<br>1.00<br>1.21 (1.07-1.37)<br>1.36 (1.13-1.63)<br>1.31 (1.02-1.68)	Age, race, diabetes, calcium supplement use, HRT, income, region, water, sodium, animal protein, dietary calcium<br>+ physical activity, energy intake
Yoshimura E et al, 2016, Japan	Natural Gas Company in metropolitan Tokyo	1985-2004/ 2005, 19 years follow-up	4074 men, age 20-40 years: 258 cases	Measured	BMI	15.9-21.6<br>21.7-23.7<br>23.8-35.6	1.00<br>1.28 (0.93-1.76)<br>1.41 (1.02-1.97)	Age, alcohol, SBP, cardiorespiratory fitness, cigarette smoking
Shu X et al, 2017, China	Shanghai Women's Health Study	1996-2000 - 2004-2008, 10 years follow-up	69166 women, age 40-70 years: 1451 cases	Measured	BMI	<18.5<br>18.5-24.9<br>25.0-29.9<br>≥30.0	1.01 (0.75-1.37)<br>1.00<br>1.14 (1.01-1.28)<br>1.17 (0.92-1.49)	Birth year, pack-years of smoking, education, income, energy intake, dietary protein intake, dietary calcium intake, calcium supplement use, vitamin C intake, vitamin C supplement use, dietary potassium intake, dietary magnesium intake, physical activity, history of coronary heart disease/stroke, type 2 diabetes, hypertension, cholelithiasis, WHR
Shu X et al, 2017, China	Shanghai Men's Health Study	2002-2006 - 2004-2008, 5 years	58054 men, age 40-74 years: 1202 cases	Measured	BMI	<18.5<br>18.5-24.9<br>25.0-29.9<br>≥30.0	0.82 (0.58-1.16)<br>1.00<br>1.15 (1.01-1.31)<br>1.30 (0.94-1.80)	Birth year, pack-years of smoking, education, income, energy intake, dietary protein intake, dietary calcium intake,

		follow-up						calcium supplement use, vitamin C intake, vitamin C supplement use, dietary potassium intake, dietary magnesium intake, physical activity, history of coronary heart disease/stroke, type 2 diabetes, hypertension, cholelithiasis, WHR

BMI=body mass index, HRT=hormone replacement therapy, hs-CRP=high sensitivity C-reactive protein, LDL=low-density lipoprotein, SBP=systolic blood pressure, TG=triglycerides, WHR=waist-to-hip ratio

Supplementary Table 3. Prospective studies of diabetes mellitus and kidney stones

First author, publication year, country	Study name or description	Study period	Number of participants, number of cases	Exposure	Comparison	Relative risk (95% confidence interval)	Adjustment for confounders
Taylor EN et al, 2005, USA	Health Professionals Follow-up Study	1980-2000, 20 years follow-up	45988 men, age 40-75 years: 1609 cases	Diabetes mellitus	Yes vs. no	1.29 (1.05-1.58)	Age, use of thiazide diuretics, alcohol, calcium supplement use, fluids, animal protein, calcium, magnesium, potassium, sodium, vitamin C
Taylor EN et al, 2005, USA	Nurses' Health Study 1	1991-2001, 10 years follow-up	93758 women, age 34-59 years: 1687 cases	Diabetes mellitus	Yes vs. no	1.60 (1.16-2.21)	Age, use of thiazide diuretics, alcohol, calcium supplement use, fluids, animal protein, calcium, magnesium, potassium, sodium, vitamin C
Taylor EN et al, 2005, USA	Nurses' Health Study 2	1986-2000, 14 years follow-up	101877 women, age 27-44 years: 1531 cases	Diabetes mellitus	Yes vs. no	0.81 (0.59-1.09	Age, use of thiazide diuretics, alcohol, calcium supplement use, fluids, animal protein, calcium, magnesium, potassium, sodium, vitamin C
Akoudad S, 2010, USA	Atherosclerosis Risk in Communities Study	1993-1995 - 2005, 10.8 years follow-up	12161 men and women, age 45-64 years: 971 cases	Diabetes mellitus	Yes vs. no	1.98 (1.20-3.28)	Age, sex, race, region, waist circumference, triglycerides, hypertension, uric acid, gallstones
Jakobsen AK et al, 2014, Sweden	Swedish Nationwide Cohort study	1997-2001 - 2009, 5.7 years follow-up	48211 men and women, mean age 46 years: 716 cases	Diabetes mellitus	Yes vs. no	1.08 (0.53-2.19)	Age, anti-TNF, uveitis, renal insufficiency, hypertension, inflammatory bowel disease, ischemic heart disease, psoriasis, nephrolithiasis, calcium metabolic disorders, arthritis urica/cystinuria/ hyperoxaluria, obesity
Masterson JH et al, 2015, USA	Naval Medical Center, San Diego	2000-NA, 3.45 years follow-up	52184 men and women, mean age 31 years: 702 cases	Diabetes mellitus	Yes vs. no	1.10 (0.90-1.40)	Age, sex, peripheral vascular disease, hypertension, coronary artery disease, obesity, tobacco, dyslipidemia
Shih MT et al, 2016, Taiwan	Taiwan National Health Insurance	2000-2008, 6.78 years	16670 men and women, age 22-79 years: 803 cases	Diabetes mellitus	Yes vs. no	1.04 (0.82-1.31)	Age, sex, ankylosing spondylitis, urbanization level, hypertension, hyperlipidemia, coronary heart

	Research Database	follow-up					disease, chronic kidney disease
Landgren AJ et al, 2017, Sweden	Western Sweden VEGA regional healthcare database	2006-2012, 3.9 years follow-up	168646 men and women, age >19 years: 2803 cases	Diabetes, gout patients<br>Diabetes, non-gout controls	Yes vs. no<br>Yes vs. no	1.55 (1.18-2.04)<br>1.11 (0.91-1.34)	Age, sex, hypertension, ischemic heart disease, kidney disease, obesity, calcium antagonists, thiazide diuretics, potassium-sparing medications, RAAS-inhibitors, losartan, loop diuretics, statins
Shu X et al, 2017, China	Shanghai Women's Health Study	1996-2000 - 2004-2008, 10 years follow-up	69166 women, age 40-70 years: 1451 cases	Type 2 diabetes	Yes vs. no	1.11 (0.85-1.44)	Birth year, pack-years of smoking, education, income, energy intake, dietary protein intake, dietary calcium intake, calcium supplement use, vitamin C intake, vitamin C supplement use, dietary potassium intake, dietary magnesium intake, physical activity, history of coronary heart disease/stroke, hypertension, cholelithiasis, BMI , WHR
Shu X et al, 2017, China	Shanghai Men's Health Study	2002-2006 - 2004-2008, 5 years follow-up	58054 men, age 40-74 years: 1202 cases	Type 2 diabetes	Yes vs. no	0.98 (0.77-1.26)	Birth year, pack-years of smoking, education, income, energy intake, dietary protein intake, dietary calcium intake, calcium supplement use, vitamin C intake, vitamin C supplement use, dietary potassium intake, dietary magnesium intake, physical activity, history of coronary heart disease/stroke, hypertension, cholelithiasis, BMI, WHR

BMI=body mass index, RAAS=renin-angiotensin-aldosterone-system, TNF=tumor necrosis factor, WHR=waist-to-hip ratio

Supplementary Table 4. Prospective studies of physical activity and kidney stones

First author, publication year, country	Study name or description	Study period	Number of participants, number of cases	Exposure	Comparison	Relative risk (95% confidence interval)	Adjustment for confounders
Sorensen MD et al, 2014, USA	Women's Health Initiative	1993-1998 - NA, 8 years follow-up	84225 women, age 50-79 years: 2392 cases	Physical activity	Inactive<br>0.1-4.9 METs/wk<br>5.0-9.9<br>10.0-19.9<br>20.0-29.9<br>≥30.0	1.00<br>0.84 (0.74-0.97)<br>0.78 (0.68-0.90)<br>0.69 (0.60-0.79)<br>0.70 (0.59-0.83)<br>0.69 (0.58-0.83)	Age, race, diabetes, calcium supplement use, HRT, income, region, water, sodium, animal protein, dietary calcium, energy intake
Ferraro PM et al, 2015, USA	Health Professionals Follow-up Study	1986-2006, 20 years follow-up	44964 men, age 40-75 years: 1841 cases	Physical activity	<5 METs/wk<br>5-9.9<br>10-19.9<br>20-29.9<br>≥30.0	1.00<br>0.96 (0.81-1.15)<br>1.03 (0.88-1.19)<br>0.97 (0.82-1.15)<br>1.00 (0.87-1.14)	Age, race, BMI, diabetes, calcium supplement use, geographic region, total energy, calcium, sodium, animal protein, fluids, caffeine, potassium, magnesium, vitamin C, fructose, oxalate, phytate, alcohol, high blood pressure, gout, profession, thiazide
Ferraro PM et al, 2015, USA	Nurses' Health Study 1	1986-2006, 20 years follow-up	74551 women, age 30-55 years: 1439 cases	Physical activity	<5 METs/wk<br>5-9.9<br>10-19.9<br>20-29.9<br>≥30.0	1.00<br>0.99 (0.85-1.15)<br>0.95 (0.82-1.10)<br>0.89 (0.74-1.08)<br>1.01 (0.85-1.19)	Age, race, BMI, diabetes, calcium supplement use, postmenopausal hormone use, geographic region, total energy, calcium, sodium, animal protein, fluids, caffeine, potassium, magnesium, vitamin C, fructose, oxalate, phytate, alcohol, high blood pressure, gout, thiazide
Ferraro PM et al, 2015, USA	Nurses' Health Study 2	1991-2007, 16 years follow-up	95618 women, age 25-42 years: 2075 cases	Physical activity	<5 METs/wk<br>5-9.9<br>10-19.9<br>20-29.9<br>≥30.0	1.00<br>1.01 (0.89-1.15)<br>0.97 (0.86-1.10)<br>0.98 (0.84-1.14)<br>1.03 (0.90-1.18)	Age, race, BMI, diabetes, calcium supplement use, postmenopausal hormone use, geographic region, total energy, calcium, sodium, animal protein, fluids, caffeine, potassium, magnesium, vitamin C, fructose, oxalate, phytate, alcohol, high blood pressure, gout, thiazide

BMI=body mass index, HRT=hormone replacement therapy, MET=metabolic equivalent tasks, wk=week

Supplementary Table 5. Table of RRs and 95% CIs from nonlinear dose-response analysis of BMI and kidney stones

	Kidney stones
BMI	RR (95% CI)
17.5	0.95 (0.85-1.07)
20.0	1.00
22.5	1.08 (0.99-1.19)
25.0	1.21 (1.03-1.42)
27.5	1.37 (1.09-1.71)
30.0	1.57 (1.18-2.08)
32.5	1.82 (1.28-2.59)
35.0	2.14 (1.40-3.28)
37.5	2.53 (1.52-4.21)
40.0	3.02 (1.66-5.50)
$p_{nonlinearity}$	0.10
$p_{overall}$	0.009

Supplementary Table 6. Table of RRs and 95% CIs from nonlinear dose-response analysis of waist circumference and kidney stones

	Kidney stones
Waist circumference	RR (95% CI)
72.5	1.00
75.0	1.06 (1.04-1.09)
80.0	1.19 (1.11-1.27)
85.0	1.31 (1.18-1.45)
90.0	1.43 (1.26-1.62)
95.0	1.54 (1.34-1.77)
100.0	1.63 (1.41-1.89)
105.0	1.69 (1.46-1.96)
110.0	1.72 (1.47-2.00)
115.0	1.71 (1.43-2.03)
120.0	1.65 (1.31-2.07)
$p_{nonlinearity}$	0.03
$p_{overall}$	<0.0001

Supplementary Table 7. Table of RRs and 95% CIs from nonlinear dose-response analysis of weight and kidney stones

	Kidney stones
Weight	RR (95% CI)
60	1.00
65	1.13 (1.09-1.18)
70	1.25 (1.18-1.33)
75	1.35 (1.26-1.45)
80	1.44 (1.34-1.55)
85	1.52 (1.41-1.64)
90	1.59 (1.48-1.71)
95	1.65 (1.53-1.78)
100	1.70 (1.58-1.84)
105	1.75 (1.61-1.91)
110	1.79 (1.63-1.97)
$p_{nonlinearity}$	<0.0001
$p_{overall}$	<0.0001

Supplementary Table 8. Table of RRs and 95% CIs from nonlinear dose-response analysis of weight gain and kidney stones

	Kidney stones
Weight gain	RR (95% CI)
+1 kg	1.00
+5	1.07 (0.98-1.18)
+10	1.16 (0.99-1.35)
+15	1.27 (1.05-1.55)
+20	1.47 (1.17-1.85)
+22.5	1.62 (1.28-2.07)
$p_{nonlinearity}$	0.12
$p_{overall}$	<0.0001

Supplementary Table 9. Table of RRs and 95% CIs from nonlinear dose-response analysis of physical activity and kidney stones

	Kidney stones
Physical activity	RR (95% CI)
0	1.00
5	0.80 (0.71-0.89)
10	0.77 (0.65-0.93)
15	0.77 (0.60-0.99)
20	0.77 (0.55-1.07)
25	0.77 (0.51-1.16)
30	0.77 (0.47-1.26)
35	0.77 (0.43-1.38)
$p_{nonlinearity}$	<0.0001
$p_{overall}$	<0.0001

Supplementary Figure 1. Influence analysis of BMI and kidney stones

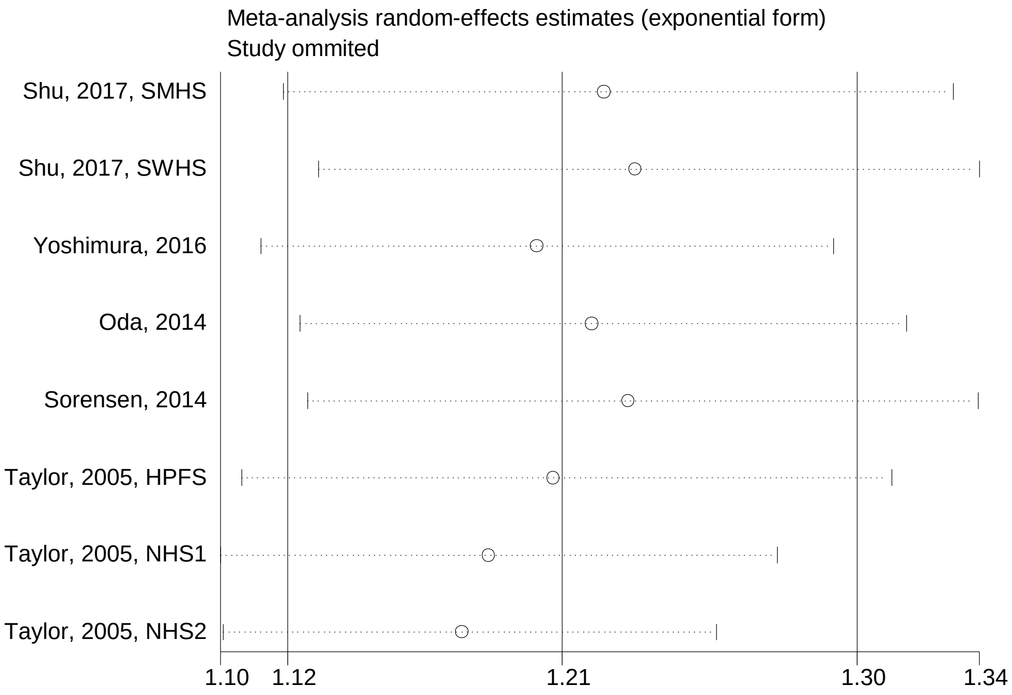

Study omitted	e^coef.	[95% Conf.	Interval]
Shu, 2017, SMHS	1.2184144	1.1181823	1.3276312
Shu, 2017, SWHS	1.2281264	1.1291627	1.3357637
Yoshimura, 2016	1.1973314	1.1112378	1.2900952
Oda, 2014	1.214574	1.1235081	1.3130212
Sorensen, 2014	1.226064	1.1257808	1.3352802
Taylor, 2005, HPFS	1.2024806	1.1051182	1.3084207
Taylor, 2005, NHS1	1.1824367	1.0986153	1.2726533
Taylor, 2005, NHS2	1.1740136	1.0995106	1.253565
Combined	1.2053105	1.1195665	1.2976214

Supplementary Figure 2. Funnel plot of BMI and kidney stones

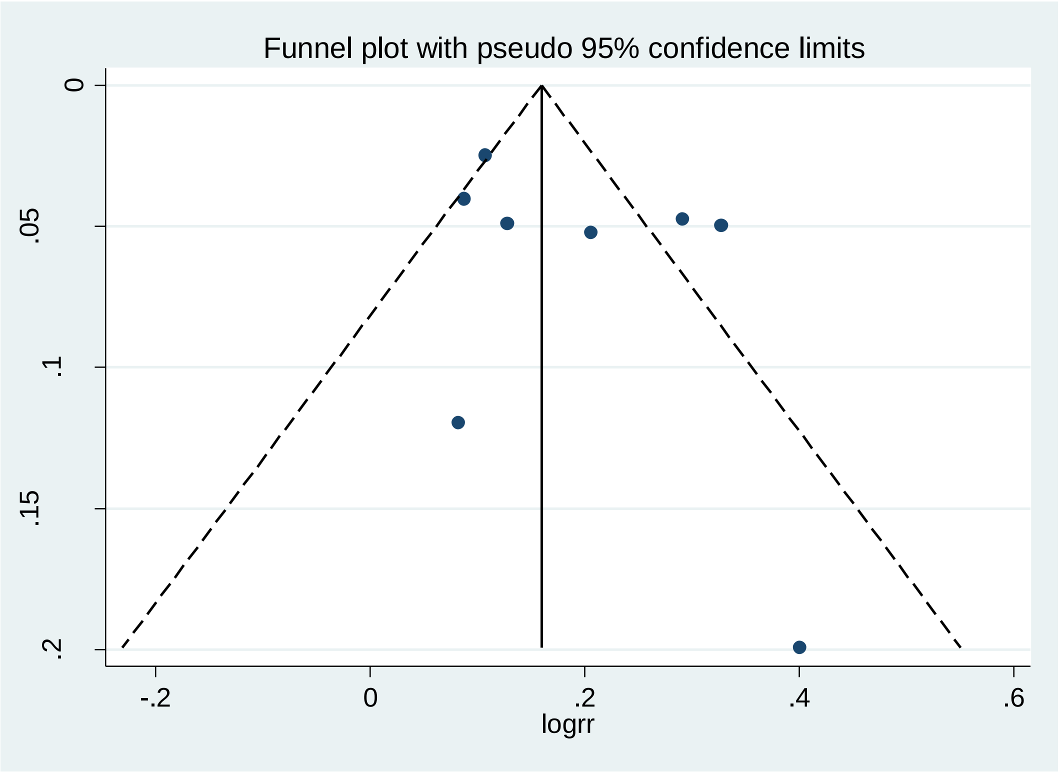

Supplementary Figure 3. Influence analysis of waist circumference and kidney stones

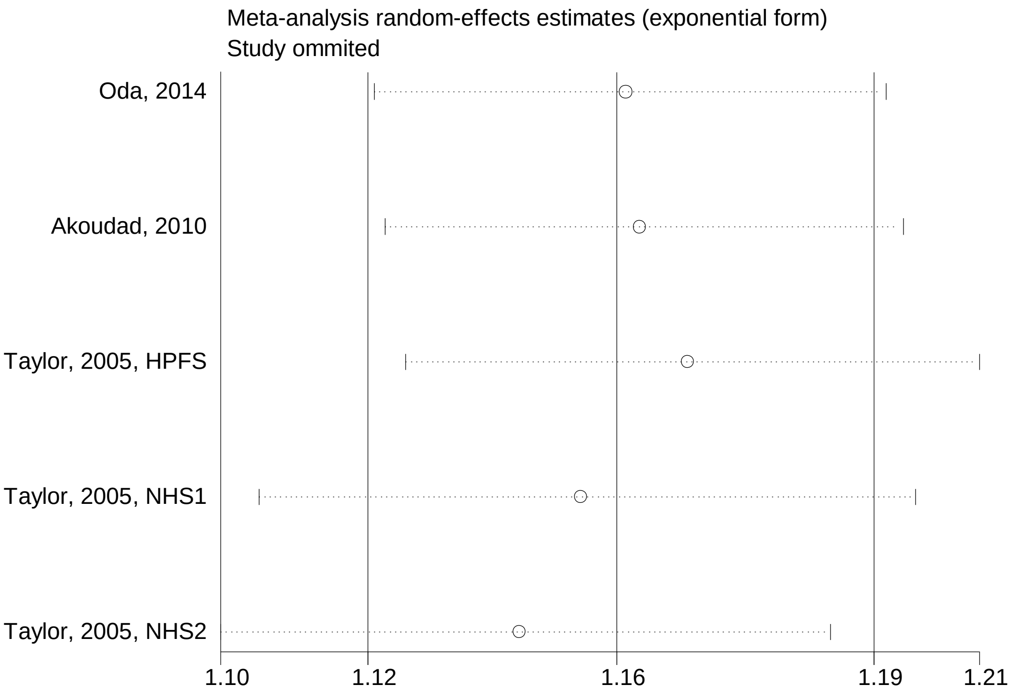

Study omitted	e^coef.	[95% Conf.	Interval]
Oda, 2014	1.1563793	1.1198394	1.1941117
Akoudad, 2010	1.1584125	1.1214253	1.1966197
Taylor, 2005, HPFS	1.1653341	1.1244533	1.2077012
Taylor, 2005, NHS1	1.1498092	1.1031824	1.1984068
Taylor, 2005, NHS2	1.1409655	1.0975747	1.1860715
Combined	1.1550535	1.1188871	1.1923889

Supplementary Figure 4. Influence analysis of diabetes mellitus and kidney stones

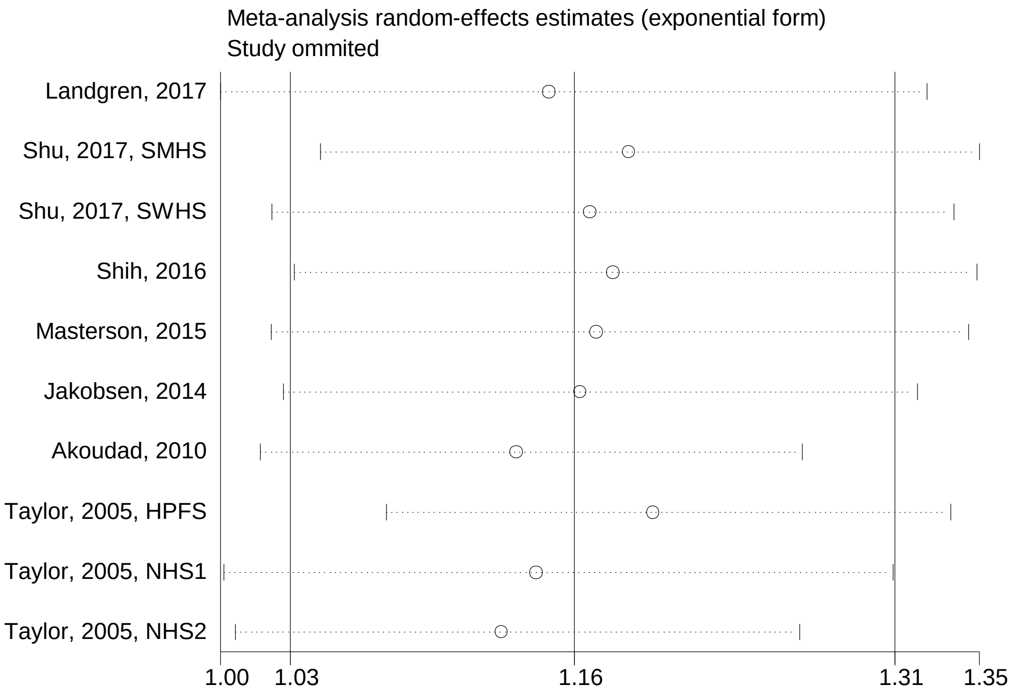

Study omitted	e^coef.	[95% Conf.	Interval]
Landgren, 2017	1.1466128	0.99516231	1.3211118
Shu, 2017, SMHS	1.1835815	1.0411488	1.3454993
Shu, 2017, SWHS	1.1656044	1.0186979	1.3336962
Shih, 2016	1.1762314	1.0292715	1.3441743
Masterson, 2015	1.1684055	1.0185137	1.3403566
Jakobsen, 2014	1.161244	1.0240749	1.3167864
Akoudad, 2010	1.1316855	1.013512	1.2636379
Taylor, 2005, HPFS	1.1947714	1.0716271	1.3320665
Taylor, 2005, NHS1	1.1407135	0.99673223	1.3054934
Taylor, 2005, NHS2	1.1247209	1.0019338	1.2625555
Combined	1.1584247	1.0272435	1.306358

c

Supplementary Figure 5. Funnel plot of diabetes mellitus and kidney stones

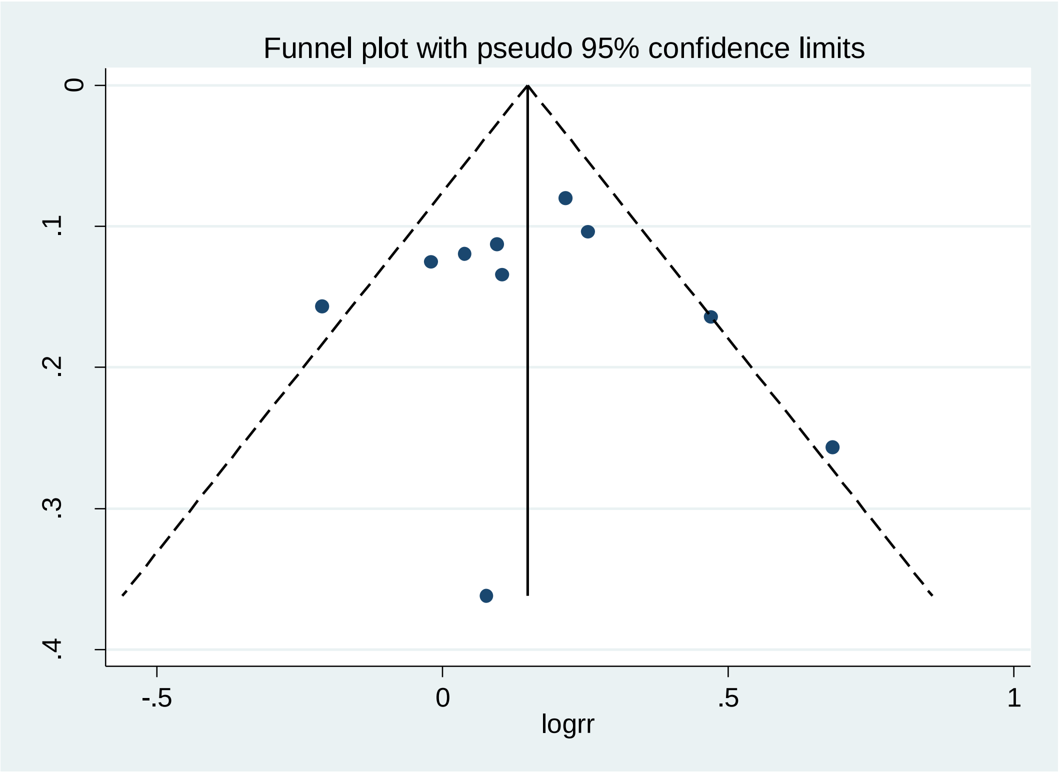

Supplement: Supplementary file 1 — Supplementary material 1 (PDF 340 kb) [file 10654_2018_426_MOESM1_ESM.pdf]
